# Correction: Effectiveness of the med Safety mobile application in improving adverse drug reaction reporting by healthcare professionals in Uganda: a protocol for a pragmatic cluster-randomised controlled trial

**DOI:** 10.1136/bmjopen-2022-061725corr1

**Published:** 2024-06-18

Kiguba R, Mwebaza N, Ssenyonga R, et al. Effectiveness of the Med Safety mobile application in improving adverse drug reaction reporting by healthcare professionals in Uganda: a protocol for a pragmatic cluster-randomised controlled trial. BMJ Open 2022;12:e061725. doi: 10.1136/bmjopen-2022-061725

This article was previously published with an error.

The protocol mistakenly indicated that the study will use block randomisation (instead of simple randomisation) to assign clusters. This error went unnoticed and is contrary to the simple randomisation approach implemented by the study. This has been corrected in *Concealment of sequence generation* and *Randomisation* section of *Assignment of interventions* under *Methods and analysis*.

