# Supplementary material for: Effectiveness of the Med Safety mobile application in improving adverse drug reaction reporting by healthcare professionals in Uganda: a protocol for a pragmatic cluster-randomised controlled trial
Source: BMJ Open. 2022 Jul 1;12(7):e061725. doi: 10.1136/bmjopen-2022-061725 (PMC9252195; doi:10.1136/bmjopen-2022-061725)
Supplement: Supplementary data [file bmjopen-2022-061725supp002.pdf]

Supplementary file 2: Baseline questionnaires for healthcare professionals and facilities

Study Questionnaire for Healthcare Professionals

Research Assistant: ______________________ Code: [ ]

Region: _________________________________ Code: [ ]

Health Facility Name: ______________________________ Code: [ ]

Date of Interview: ____/_____/_____

Adverse drug reaction (ADR) is an unwanted or harmful reaction experienced following the administration of a drug or combination of drugs under normal doses/conditions of use & is suspected to be related to the drug. An ADR might require the drug to be discontinued or the dose reduced.

SECTION A: DEMOGRAPHICS

1. Gender:
   [1] Male
   [2] Female
2. Age (*in completed years*): _____
3. Do you own a working smartphone?
   [1] Yes
   [2] No

   3a) If ***YES*** to **Q3**, are you willing to join a WhatsApp group? [1] Yes [2] No

   3b) If ***YES*** to **Q3a**, can you share your WhatsApp number? _____________
4. Do you own a simple working mobile phone *(not a smartphone)*?
   [1] Yes
   [2] No
5. Highest Education Level:
   [1] Certificate
   [2] Diploma
   [3] Bachelors
   [4] Masters
   [5] Other, (*specify*)______________
6. Professional experience (*Years*): [ ]
7. If less than 1 year in **Q5**, state number of *completed months* _____
8. Do you use any of the following? (***Tick all that apply***)
   [1] WhatsApp
   [2] Facebook
   [3] Twitter
   [4] Other, (*specify*) _____________
9. Professional Cadre:
   [1] Physician
   [2] Medical Officer
   [3] Pharmacist
   [4] Nurse/Midwife
   [5] Clinical Officer
   [6] Pharmacy Technician
   [7] Lay Counsellor
   [8] Expert Client
   [9] Other, (*specify*) _____________
10. Health Facility Type:
    [1] Public
    [2] Private Not-for-Profit
    [3] Private for-Profit
11. Health Facility Status:
    [1] Regional Referral Hospital
    [2] Hospital
    [3] Health Centre IV
    [4] Health Centre III
    [5] Health Centre II
    [6] Private Clinic
    [7] Other, (*specify*) _____________

Kiguba R, *et al. BMJ Open* 2024; 12:e061725. doi: 10.1136/bmjopen-2022-061725

SECTION B: DOLUTEGRAVIR-LINKED AND ISONIAZID-LINKED ADRS

Please, complete the questionnaire by indicating the appropriate responses.

1. What is the approximate number of HIV-positive patients you see per day? _____

Dolutegravir-linked adverse drug reactions

2. Dolutegravir-based antiretroviral therapy was recently rolled out in the treatment of HIV-positive patients, have you ***ever encountered*** any dolutegravir-linked adverse drug reactions (ADRs) amongst the HIV-positive patients in your care?
   [1] Yes [2] No [9] I don't know

3. In the past 4-weeks, have you ***suspected*** any dolutegravir-linked ADRs?
   [1] Yes [2] No (***Skip to 5***) [9] I don't know (***Skip to 5***)

4. If ***YES*** to **Q3**, how many cases of dolutegravir-linked ADRs have you ***suspected*** in the past 4-weeks? _____

5. In the past 4-weeks, have you ***received*** any ***patient-complaints*** of dolutegravir-linked ADRs?
   [1] Yes [2] No (***Skip to 7***) [9] I don't know (***Skip to 7***)

6. If ***YES*** to **Q5**, how many ***patient-complaints*** of dolutegravir-linked ADRs have you ***received*** in the past 4-weeks? _____

7. Briefly describe the most recent ***dolutegravir-linked*** ADR you have encountered giving details on ***patient age***, ***sex***, ***approximate ADR-date, details of the adverse drug reaction***, ***severity*** *(mild, moderate, severe)*, ***seriousness*** (*led to or prolonged hospitalization, caused incapacitation, led to any other medically significant condition or death*), ***clinical outcome*** & ***action taken***:

   Patient age [ ] years; **sex** [M or F]; **approximate ADR-date** [*mm/yyyy*: __ / ____]

   ADR description (*include severity, seriousness, clinical outcome and action taken*)____
   ____
   ____
   ____
   ____
   ____

Isoniazid preventive therapy-linked adverse drug reactions

8. HIV-positive patients are at a higher risk of dying when they develop active tuberculosis (TB). During the past one year, Ministry of Health scaled up the roll-out of isoniazid preventive therapy (IPT) among HIV-positive patients to significantly reduce these patients' risk of developing active TB. IPT, however, has been associated with ADRs. Have you ***ever encountered*** any IPT-linked ADRs amongst the HIV-positive patients in your care?
   [1] Yes [2] No [9] I don't know

9. In the past 4-weeks, have you ***suspected*** any isoniazid preventive therapy-linked ADRs?
   [1] Yes [2] No (***Skip to 11***) [9] I don't know (***Skip to 11***)

Kiguba R, *et al. BMJ Open* 2024; 12:e061725. doi: 10.1136/bmjopen-2022-061725

10. If ***YES*** to **Q9**, how many isoniazid preventive therapy-linked ADRs have you ***suspected*** in the *past 4-weeks*? _____

11. In the past 4-weeks**,** have you ***received*** any ***patient-complaints*** of isoniazid preventive therapy-linked ADRs?

    [1] Yes [2] No (***Skip to 13***) [9] I don't know (***Skip to 13***)

12. If ***YES*** to **Q11**, how many ***patient-complaints*** of isoniazid preventive therapy-linked ADRs have you ***received*** in the past 4-weeks? _____

13. Briefly describe the most recent ***isoniazid-linked*** ADR you have encountered giving details on ***patient age***, ***sex***, ***approximate ADR-date, details of the adverse drug reaction***, ***severity*** *(mild, moderate, severe)*, ***seriousness*** (*led to or prolonged hospitalization, caused incapacitation, led to any other medically significant condition or death*), ***clinical outcome*** & ***action taken***:

    Patient age [ ] years; **sex** [M or F]; **approximate ADR-date** [*mm/yyyy*: __ / ____]

    ADR description (*include severity, seriousness, clinical outcome and action taken*)____
    ____
    ____
    ____
    ____
    ____

14. In the past 6-months, have you ***reported*** any suspected ADRs experienced by the HIV-positive patients under your care?

    [1] Yes [2] No (***Skip to Q23***) [9] I don't know (***Skip to Q23***)

15. If ***YES*** to **Q14**, how many ***ADR reports*** did you make? [ ][ ]

16. If ***YES*** to **Q14**, which drug classes were suspected? (***Tick all that apply***)

    [1] Antiretroviral therapy

    [2] Isoniazid preventive therapy

    [3] Antituberculosis therapy

    [4] Other, (*specify*)____

17. If ***YES*** to **Q14**, which of the following drugs were implicated? (***Tick all that apply and mention number of reported ADRs in past 4-weeks for options [1] & [2]***)

    [1] Dolutegravir: *Number of ADRs* ***reported*** *in the past 4-weeks* _____

    [2] Isoniazid: *Number of ADRs* ***reported*** *in the past 4-weeks* _____

    [3] Other, (*specify*) ______________

18. If ***YES*** to **Q14**, to whom did you report the most recent ADR? (***Tick all that apply***)
    [1] District Health Officer
    [2] Health Management Information System
    [3] Immediate Supervisor
    [4] National Drug Authority - National Pharmacovigilance Center
    [5] Other, (*specify*)__________________

19. If ***YES*** to **Q14**, how did you report the most recent suspected ADR? (***Tick all that apply***)
    [1] Verbally
    [2] Written paper report
    [3] Written online/website report
    [4] Other, (*specify*) __________________

20. If ***YES*** to **Q14**, did you get feedback on the ADR(s) you reported?
    [1] Yes [2] No [9] Not sure

21. If ***YES*** to **Q20**, what form of feedback?
    ____
    ____

22. In special situations, the National Drug Authority may need additional information about reported ADRs. Would you be willing to answer further questions after sending a report?
    [1] Yes [2] No [9] I don't know

Herbal Medicine Use

23. In the past 6-months, have you suspected that some of your patients use herbal medicines together with ARVs? [1] Yes [2] No

24. If YES to **Q23**, have you inquired from the patients whether they use herbal medicines together with ARVs? [1] Yes [2] No

25. In the past six months, have any of your patients reported to you that they use herbal medicines together with ARVs? [1] Yes [2] No

26. Have any of your patients presented with ADRs likely to be linked to the use of herbal medicines? [1] Yes [2] No

We appreciate the time you've taken to respond to this survey. Thank you!

Kiguba R, *et al. BMJ Open* 2024; 12:e061725. doi: 10.1136/bmjopen-2022-061725

Health Facility Data

Research Assistant: ____________________ Code: [ ]

Region: ______________________________ Code: [ ]

Health Facility Name: ____________________________ Code: [ ]

Date of Enrolment of Health Facility: ____/_____/_____

Average daily number of ART patient visits at the Health Facility: [ ][ ][ ]

Total number of patients on dolutegravir-containing antiretroviral therapy [ ][ ][ ][ ]

Total Number of patients on Isoniazid Preventive Therapy [ ][ ][ ][ ]

Number of healthcare professionals at the Health Facility:

- Medical Doctors [ ][ ]
- Pharmacists [ ][ ]
- Clinical Officers [ ][ ]
- Nurses/Midwives [ ][ ]
- Pharmacy Technicians [ ][ ]
- Village Health Teams [ ][ ]
- Lay Counsellors [ ][ ]
- Expert Clients [ ][ ]
- Other (*specify*) ……………………….. [ ][ ]

Does the Health Facility provide health workers with internet access?
1. No 2. Yes 9. Unknown

If YES, describe the form of internet access provided (*wireless, wired, data etc.*):

____

____

Is mobile telephone network connectivity available at the Health Facility?
1. No 2. Yes 9. Unknown

If YES, describe the reliability of mobile telephone network connectivity:

____

____

Kiguba R, *et al. BMJ Open* 2024; 12:e061725. doi: 10.1136/bmjopen-2022-061725
