# Supplementary material for: Effectiveness of the Med Safety mobile application in improving adverse drug reaction reporting by healthcare professionals in Uganda: a protocol for a pragmatic cluster-randomised controlled trial
Source: BMJ Open. 2022 Jul 1;12(7):e061725. doi: 10.1136/bmjopen-2022-061725 (PMC9252195; doi:10.1136/bmjopen-2022-061725)
Supplement: Supplementary data [file bmjopen-2022-061725supp003.pdf]

Informed Consent Form

Purpose of the study: Experts from National Drug Authority and Makerere University College of Health Sciences are working together to improve the rate and quality of reporting of adverse drug reactions linked to the newly rolled-out dolutegravir-containing antiretroviral therapy and scaled up isoniazid preventive therapy to reduce the risk of tuberculosis in HIV-positive people. This project focuses on the involvement of healthcare professionals in the active drug safety monitoring and management of adverse drug reactions to these drugs and how this system can be improved.

Procedures: In this survey, we ask you to recall and describe the most recent suspected and/or reported adverse drug reactions (ADRs) linked to the use of dolutegravir-containing antiretroviral therapy and isoniazid preventive therapy among the HIV-positive people in your care; and any ADRs you have suspected in the past 4-weeks and/or ADR-complaints you have received from HIV patients in the past 4-weeks. The survey is anonymous and will take no more than 20-30 minutes to complete. After the interview, we shall provide you with the tools you will use to report any adverse drug reactions you encounter, particularly those linked to the use of dolutegravir and isoniazid preventive therapy. You will be contacted by phone at the end of the study to obtain feedback from you about your experience while using the tools for reporting adverse drug reactions. You could even be invited to participate in a group or individual in-depth discussion to share your experiences.

Time compensation: We greatly appreciate your time and opinion. The time you spend filling in this questionnaire will be compensated at a rate of UGX 20,000.

Confidentiality: Any information obtained in this study that can identify you will remain confidential and be disclosed only with your permission or as required by law. Otherwise, confidentiality will be maintained because the self-completed questionnaire is anonymous. We will not request for your name in the information we get from this study.

Participation and withdrawal: Participation is voluntary and you can withdraw at any time.

Identification of Investigators: If you have any questions or concerns about the research, please feel free to contact: Dr. Ronald Kiguba, Principal Investigator, Department of Pharmacology & Therapeutics, School of Biomedical Sciences, Makerere University College of Health Sciences, Tel: +256712840683 & email: kiguba@yahoo.co.uk

Rights of participants: The School of Biomedical Sciences Research & Ethics Committee and the Uganda National Council for Science & Technology (UNCST) have reviewed my request to conduct this project. If you have any concerns about your rights in this study, please contact Dr. Erisa Mwaka Tel: +256 752575050 & email: erisamwaka@gmail.com OR UNCST on +256 414 705500 / +256 312 314800

I understand the procedures described above. My questions have been answered to my satisfaction, and I agree to participate in this study. I have been given a copy of this form.

Signature of participant: ……………………………… Date: ……………………………………

Signature of research assistant: ………………………. Date: ……………………………………
