# Supplementary material for: Effectiveness of the Med Safety mobile application in improving adverse drug reaction reporting by healthcare professionals in Uganda: a protocol for a pragmatic cluster-randomised controlled trial
Source: BMJ Open. 2022 Jul 1;12(7):e061725. doi: 10.1136/bmjopen-2022-061725 (PMC9252195; doi:10.1136/bmjopen-2022-061725)
Supplement: Author's manuscript [file bmjopen-2022-061725.draft_revisions.pdf]

BMJ Open

BMJ Open is committed to open peer review. As part of this commitment we make the peer review history of every article we publish publicly available.

When an article is published we post the peer reviewers' comments and the authors' responses online. We also post the versions of the paper that were used during peer review. These are the versions that the peer review comments apply to.

The versions of the paper that follow are the versions that were submitted during the peer review process. They are not the versions of record or the final published versions. They should not be cited or distributed as the published version of this manuscript.

BMJ Open is an open access journal and the full, final, typeset and author-corrected version of record of the manuscript is available on our site with no access controls, subscription charges or pay-per-view fees (http://bmjopen.bmj.com).

If you have any questions on BMJ Open's open peer review process please email info.bmjopen@bmj.com

BMJ Open

Effectiveness of the Med Safety mobile application in improving adverse drug reaction reporting by healthcare professionals in Uganda: a protocol for a pragmatic cluster-randomized controlled trial

Journal:	*BMJ Open*
Manuscript ID	bmjopen-2022-061725
Article Type:	Protocol
Date Submitted by the Author:	04-Feb-2022

Complete List of Authors:	Kiguba, Ronald; Makerere University College of Health Sciences, Pharmacology and Therapeutics<br>Mwebaza, Norah; Makerere University College of Health Sciences, Pharmacology and Therapeutics<br>Ssenyonga, Ronald ; Makerere University, Epidemiology & Biostatistics<br>Ndagije, Helen ; National Drug Authority, National Pharmacovigilance Centre<br>Nambasa, Victoria; National Drug Authority, National Pharmacovigilance Centre<br>Katureebe, Cordelia; Ministry of Health<br>Katumba, Kenneth; MRC/UVRI and LSHTM Uganda Research Unit<br>Tregunno, Phil; Medicines and Healthcare Products Regulatory Agency<br>Harrison, Kendal; Medicines and Healthcare Products Regulatory Agency<br>Karamagi, Charles; Makerere University College of Health Sciences, Clinical Epidemiology Unit, Department of Medicine<br>Scott, Kathryn A.; University of Liverpool, MRC Centre for Drug Safety Science and Wolfson Centre for Personalised Medicine, Institute of Systems, Molecular and Integrative Biology (ISMIB)<br>Pirmohamed, Munir; University of Liverpool, MRC Centre for Drug Safety Science and Wolfson Centre for Personalised Medicine, Institute of Systems, Molecular and Integrative Biology (ISMIB)

Keywords:	Adverse events < THERAPEUTICS, CLINICAL PHARMACOLOGY, EPIDEMIOLOGY, Epidemiology < INFECTIOUS DISEASES

SCHOLARONE™ Manuscripts

Effectiveness of the Med Safety mobile application in improving adverse drug reaction reporting by healthcare professionals in Uganda: a protocol for a pragmatic cluster-randomized controlled trial

Authors:

Ronald Kiguba[1], Norah Mwebaza[1], Ronald Ssenyonga[2], Helen B. Ndagije[3], Victoria Nambasa[3], Cordelia Katureebe[4], Kenneth R. Katumba[5], Phil Tregunno[6], Kendal Harrison[6], Charles Karamagi[7], Kathryn A. Scott[8], Munir Pirmohamed[8]*

*Correspondence to: Ronald Kiguba (Tel: +256 701 840683; Email: kiguba@gmail.com)

Affiliations:

[1]Department of Pharmacology and Therapeutics, College of Health Sciences, Makerere University, Kampala, Uganda

[2]Department of Epidemiology and Biostatistics, College of Health Sciences, Makerere University, Kampala, Uganda

[3]National Pharmacovigilance Centre, National Drug Authority, Kampala, Uganda

[4]AIDS Control Programme, Ministry of Health, Kampala, Uganda

[5]MRC/UVRI & LSHTM Uganda Research Unit

[6]Medicines and Healthcare products Regulatory Agency, London, United Kingdom

[7]Clinical Epidemiology Unit, College of Health Sciences, Makerere University, Kampala, Uganda

[8]MRC Centre for Drug Safety Science and Wolfson Centre for Personalised Medicine, Institute of Systems, Molecular and Integrative Biology (ISMIB), University of Liverpool, Liverpool, United Kingdom

For peer review only

For peer review only - http://bmjopen.bmj.com/site/about/guidelines.xhtml

Abstract

Introduction: Combination antiretroviral therapy (cART) has massively reduced HIV mortality. However, long-term cART increases the risk of adverse drug reactions (ADRs), which can lead to higher morbidity, mortality, and healthcare costs for people living with HIV (PLHIV).

Pharmacovigilance – monitoring the effects of medicines – is essential for understanding real-world drug safety. In Uganda, pharmacovigilance systems have only recently been developed and rates of ADR-reporting for cART are very low. Thus, the risk profile of medicines currently used to treat HIV and tuberculosis in our population is poorly understood.

The Med Safety mobile application has been developed through the European Union's Innovative Medicines Initiative WEB-Recognising Adverse Drug Reactions project to promote digital pharmacovigilance. This mobile application has been approved for ADR-reporting by Uganda's National Drug Authority. However, the barriers and facilitators to Med Safety uptake, and its effectiveness in improving pharmacovigilance are as yet unknown.

Methods and analysis: A pragmatic cluster-randomized controlled trial will be implemented over 30-months at 191 intervention and 191 comparison cART-sites to evaluate Med Safety. Using a randomization sequence generated by the sealed envelope software, we shall randomly assign the 382 pre-screened cART-sites to the intervention and comparison arms. Each cART-site is a cluster which consists of healthcare professionals and PLHIV receiving dolutegravir (DTG)-based ART and/or Isoniazid Preventive Therapy. Healthcare professionals enrolled in the intervention arm will be trained in the use of mobile-, paper- and web-based reporting, whilst those in the comparison arm will be trained in paper- and web-based reporting only.

Ethics and dissemination: Ethical approval was given by the School of Biomedical Sciences Research and Ethics Committee at Makerere University (SBS-REC-720) and administrative clearance obtained from Uganda National Council for Science and Technology (HS1366ES). Study results will be shared with healthcare professionals, policy-makers, the public and academia.

Registration: PACTR202009822379650.

For peer review only

For peer review only - http://bmjopen.bmj.com/site/about/guidelines.xhtml

Strengths and limitations of the study

- This study demonstrates that collaboration between researchers, policymakers, software developers, healthcare providers and consumers can be achieved to promote medication safety in resource-limited settings.
- The study results will be readily available for immediate use to inform policy and practice by key stakeholders including healthcare providers, drug regulators, researchers, Med Safety mobile app developers and users, and policymakers.
- We aim to recruit 3,820 healthcare professionals from 382 antiretroviral therapy (ART)-sites spread across Uganda; the selected study sites serve 80% of people living with HIV receiving combination ART.
- A limitation is that the use of the Med Safety mobile app requires a smartphone but only 16% of Ugandans own a smartphone; limited rollout showed that 7 in 10 healthcare professionals are smartphone owners.

For peer review only

For peer review only - http://bmjopen.bmj.com/site/about/guidelines.xhtml

Introduction

Spontaneous adverse drug reaction (ADR)-reporting is the backbone of pharmacovigilance (PV) globally but is plagued by under-reporting.[1-3] Only 3% of Ugandan healthcare professionals (HCPs) reported a suspected ADR to the National Pharmacovigilance Centre (NPC) in 2014.[2] These HCPs cited lack of conventional paper-based ADR-forms (paper-forms), limited access to web-based forms (web-forms) due to the scarcity of internet-wired computers, and the lack of feedback to ADR-reporters as common barriers to PV. Also, logistical challenges lurk in the countrywide distribution and collection of paper-forms by Uganda's NPC, which hinders timely data collation and analysis.[2] Ultimately, the implementation of requisite policy actions to safeguard the public from medication-related harm has been delayed.

Innovative interventions are needed to improve ADR-reporting, such as the use of mobile applications (mobile apps or apps) to support traditional PV.[4] Mobile apps have had variable success in PV worldwide. A 10-fold increase in the rate of ADR-reporting was seen for a medical device with social media engagement in the USA. In contrast, uptake of mobile app reporting has been poor in Europe: studies in the United Kingdom, the Netherlands and Croatia showed 1.7, 1.1 and 6.5 app-ADR-reports per completed-month per 1,000 app-downloads, respectively.[4,5] Uptake was also poor in India: 4.0 app-ADR-reports per completed-month per 1,000 app-downloads.[6]

Efforts to improve PV in people living with HIV (PLHIV) in Uganda are timely due to the recent rollout of dolutegravir (DTG) as the preferred first-line antiretroviral therapy (ART) for PLHIV as part of combination therapy (cART). DTG is more effective and has a higher genetic barrier to developing drug resistance than other ARTs.[7] Rollout began in October 2018 and, by August 2019, over 347,888 PLHIV were receiving DTG-based cART.[7] In addition, Uganda is rapidly scaling up Isoniazid Preventive Therapy (IPT) for the prevention of tuberculosis (TB) in PLHIV. Only 16% of PLHIV had been reached with life-saving IPT by June 2019:[8] an extra 135,711 PLHIV were initiated on IPT by September 2019 after a 100-day scale-up campaign.[7,8] IPT significantly reduces the incidence of active TB, curbing TB-related mortality among PLHIV.[9]

Although DTG is generally well tolerated, it is associated with serious ADRs including hyperglycaemia (up to 14% of patients),[10] neuropsychiatric effects (1.7%)[11] and hepatotoxicity (0.1%)[12] in European populations. DTG is also associated with more common, but less serious, ADRs such as headache, abnormal dreams and abdominal pain, but the impact of these has not been evaluated in real-world settings in developing countries. The rapid rollout of DTG and scale up of IPT will increase the number of PLHIV exposed to these therapies, potentially increasing the incidence of ADRs, including serious ADRs, associated with these medicines. Discontinuation of DTG and/or IPT is recommended if serious ADRs such as jaundice, blurred vision or hyperglycaemia occur.[13] Four in 100,000 PLHIV who receive IPT die due to an IPT-related adverse event.[1]

Adverse drug reactions can reduce quality of life for PLHIV, and increase morbidity, mortality, and healthcare costs.[14,15] In 2016, prior to DTG rollout, the NPC attempted to improve spontaneous ADR-reporting via a web-form. However, they received only 92 online reports versus 290 paper-based ADR-reports linked to ART from October 2018 to September 2019. More recently, a PV task-force comprised of the NPC, the AIDS Control Program and Ministry of Health (MoH) was set up. The task-force established an active drug safety monitoring and management program to complement the spontaneous ADR-reporting system. The NPC received 109 DTG-linked ADR-reports from October 2018 to September 2019 from ~348,000 PLHIV. Only 18 IPT-linked ADR-reports were received from January 2019 to June 2019 from ~300,000 PLHIV: 2.6 DTG-linked ADR-reports per month per 100,000 treated-PLHIV and 1.0 IPT-linked ADR-report per month per 100,000 treated-PLHIV. These rates are barely 5% the known incidence of the ADRs in PLHIV elsewhere.[1,3]

The Med Safety mobile application was developed by the European Union Innovative Medicines Initiative: WEB-Recognising Adverse Drug Reactions (WEB-RADR). The same technology that produced the Med Safety App was also adopted by European countries including UK's Medicines and Healthcare products Regulatory Agency (MHRA) in July 2015, Netherlands' Lareb in January 2016 and Croatia's HALMED in May 2016. In 2017 the Med Safety App was introduced in Africa (Burkina Faso, Zambia) in partnership with World Health Organization (WHO). MHRA adapted Med Safety for low and middle-income countries (LMICs) including Uganda with approval from Uganda's National Drug Authority (NDA), **Figure 1**.[4,16] Med Safety was launched in limited settings in Uganda in February 2020.

Med Safety's platform facilitates easy adoption and low-cost maintenance in LMICs. It is

For peer review only - http://bmjopen.bmj.com/site/about/guidelines.xhtml

available at no cost for mobile phones and tablets, for Android and iOS, in English. Within the app, ADR-reports can be completed offline and transmitted to NPC when internet connectivity is established. The ADR-reporting form has a clear and simple format. App-users can browse and view ADR-data and may create a "watch-list" of *medicines* of their own interest to receive personalized news and alerts. Med Safety provides for two-way exchange of medication-safety information between NPC and HCPs. This enhanced interaction promotes the involvement of HCPs in PV activities. The anticipated increase in volume of ADR-reports in the national PV database could enhance its signal detection potential,[17] and contribute to remedial efforts to improve patient safety. The app is also integrated into the WHO Collaborating Centre for International Drug Monitoring – Uppsala Monitoring Centre (WHO-UMC) application program interfaces (including Vigiflow, VigiAccess, WHODrug).[16] This study seeks to understand whether Med Safety is effective in improving ADR-reporting by HCPs if used together with traditional PV methods versus if traditional PV methods are used alone.

Aims and objectives

Our aim is to assess the feasibility, effectiveness, cost and cost-effectiveness of implementing Med Safety for HCP-driven reporting of ADRs associated with DTG-containing ART and IPT for TB prevention in PLHIV in Uganda. Our hypothesis is that the use of Med Safety for ADR-reporting by HCPs attending to PLHIV on DTG-based ART and/or IPT will increase the ADR-reporting-rate[18,19] by at least 25% versus use of existing PV methods alone in 30 months of follow-up at selected cART-sites in Uganda. We will test our hypothesis in a pragmatic multi-centre open-label cluster-randomized controlled trial whose specific objectives are to:

1. Assess the feasibility and acceptability of Med Safety for ADR-reporting by HCPs at selected ART-sites in Uganda
2. Determine the app's effect on the rate of ADR-reporting versus traditional PV methods alone
3. Estimate the app's cost and cost-effectiveness from the provider perspective

Methods and Analysis

Participants, interventions and outcomes

Study setting: The study will be conducted nationwide at 382 (of 1,832 ART-sites) high volume accredited ART-sites in Uganda. The 382 ART-sites serve 80% of the PLHIV on cART in Uganda.[8] This protocol follows the Standard Protocol Items: Recommendations for Interventional Trials 2013 statement, **supplementary file 1**.[20]

Eligibility criteria: All smartphone-owning HCPs at these sites are eligible. Limited rollout showed that 7 in 10 HCPs are smartphone owners. Written informed consent is sought from eligible HCPs. We expect to enrol 10 HCPs per ART-site on average.

Intervention arm: HCPs at intervention ART-sites will be introduced to the Med Safety mobile app whether they own a smartphone or not. Mobile app, paper-form and web-form awareness campaigns including initial face-to-face training, posters/brochures and monthly reminder WhatsApp and mobile-phone short messages (SMS) for up to 6-months will be undertaken. Training will be conducted by pharmacists from NPC and Makerere University with expertise in PV. The training teams have harmonized the training schedule to ensure uniform training. Interested HCPs with personal smartphones will be invited and assisted to install the mobile app and trained to use it to report suspected ADRs, with emphasis on DTG- and IPT-linked ADRs. HCPs will also be trained and encouraged to use traditional PV methods (paper-form, web-form).

Comparison arm: HCPs at the comparison ART-sites will be trained and encouraged to use traditional methods of ADR-reporting (paper-form, web-form). Med Safety will not be introduced to this group of HCPs. Reminder WhatsApp and SMS about the paper-form and web-form will be sent out monthly for up to 6-months.

Outcomes*:* Our primary outcome is number of HCP-reported ADRs per 100,000 person-months of

For peer review only - http://bmjopen.bmj.com/site/about/guidelines.xhtml

treated-PLHIV per study arm. Our secondary outcomes are number of app-ADR-reports per 1,000 app-downloads per month of follow-up; causality (by Naranjo Scale and Liverpool Causality Assessment Tool)[21,22]; seriousness as per the WHO definitions (threatens life i.e. leads to or prolongs hospitalization, causes incapacitation or death); ADR-outcome; cost per ADR-report; cost per additional ADR-report, cost per additional avoidable serious ADR-report.

Participant timeline: There are 15 regional referral hospitals each with a catchment of ~25 ART-sites. Each team of three RAs requires four weeks to enrol HCPs in one regional catchment. Follow-up at each ART-site begins after its enrolment and lasts 30 months. The 30-month follow-up period is considered adequate to monitor durability of the real-life impact of the app on ADR-reporting. Four additional months will be required to wrap-up the study, thus, 36-months overall, **Table 1**.

Table 1. Schedule of enrolment, interventions and assessments.

	STUDY PERIOD						
	**Baseline**	**Follow-up schedule**					**Close-out**
**Procedures**	**(t_0)**	***t_0+3 months***	***t_0+6 months***	***t_0+12 months***	***t_0+15 months***	***t_0+30 months***	***t_0+36 months***
**ENROLMENT:**							
Eligibility screen	X						
Informed consent	X						
Allocation to study arm	X						
**INTERVENTIONS:**							
Mobile App Arm	X	X	X	X	X	X	
Comparison Arm	X	X	X	X	X	X	
**ASSESSMENTS:**							
Quantitative Survey	X			X			
Mobile App Data	X	X	X	X	X	X	
Economic Cost Data	X	X	X	X			
Interim Data Analysis					X		
Qualitative Study	X			X			
Endline Data Analysis							X

Sample Size: To estimate the number of ART-sites required per arm for an effect size of 25%:[23] we assume power of 80% at the 95% confidence level, mean of 1.0 IPT-linked ADR/month/100,000 treated-PLHIV (0.00001) and standard deviation (SD) of 1.179 IPT-linked ADRs/month/100,000 treated-PLHIV (0.00001179) (SD was computed based on the monthly IPT-linked ADR-reports submitted to NPC for one-year from October 2018 to September 2019), cluster-size of 10 HCPs and

For peer review only - http://bmjopen.bmj.com/site/about/guidelines.xhtml

For peer review only

coefficient of variation of 0.25,[23] which yields ~114 ART-sites per trial-arm or 228 in both study arms. A minimum of 189 ART-sites per arm or 378 in both study arms (66% increment) will be required to cater for the following limitations; i) the large proportion of HCPs who do not own smartphones of ~50-60% as previously estimated[21, 22] – and is 26% [95% confidence interval (CI) of 23% to 30%] from limited rollout of RCT, ii) refusal to participate by eligible HCPs of ~5% as previously estimated[24] – and is 0% [95% CI of 0% to 2%] from limited rollout of RCT, iii) loss to follow-up due to the relatively long follow-up period of 30-months (~10%) and, iv) contamination due to information sharing between study arms through social media (~30%).[25] We shall enrol 382 ART-sites (191 per study arm) to cater for other unforeseen limitations; thus, recruiting up to 3,820 HCPs (1,910 HCPs per trial-arm). Limited rollout of the RCT suggests a cluster size of 7.2 (629/87) HCPs. We shall retain the average cluster size of 10 HCPs because the RCT has not yet enrolled from the central region of Uganda (with the country's capital city) where ART-sites tend to be much larger with more HCPs per site than the more rural eastern, western and northern regions.

Assignment of interventions

Unit of randomization: This unit is a cluster, defined as an ART-site located at each of the 382 pre-screened health facilities and consisting of HCPs and DTG/IPT-treated PLHIV. The use of ART-sites as clusters minimizes co-intervention, duplication of reporting, and organizational challenges, and provides valid PLHIV population denominators for analysis of the outcomes.

Concealment of sequence generation*:* Using the sealed envelope software, an independent Biostatistician from the Clinical Epidemiology Unit at Makerere University, who will not participate in the subsequent data analyses, generated the block randomization sequence to ensure balanced allocation of clusters.

Randomization: The pre-screened ART-sites will be assigned by research assistants to the intervention and comparison arms, **Figure 2**, using an electronically generated block randomization sequence.

Blinding: PLHIV, HCPs, PV assessors of the ADR-reports submitted to NPC and the Biostatistician who will analyze the data will be blinded to the allocation of ART-sites. The research assistants will not be blinded to the allocation of ART-sites due to the nature of the intervention.

Data collection

Baseline survey: Consented HCPs will complete an interviewer-administered electronic questionnaire using the Open Data Kit (ODK) software to record participants' characteristics and details on ADR-reporting. Details of the questionnaires are provided in **supplementary file 2**.

Follow-up survey: A follow-up survey will be conducted by phone interview and keyed into tablet based ODK pre-programmed electronic questionnaires in a random 5% of intervention ART-sites (~10) after one-year of follow-up. App-users will be asked to report their experiences to gauge app-feasibility, assess user-satisfaction, and identify potential app-revisions.

Mobile app data*:* The app is hosted by NDA where the NPC is located, which is also the preferred source of ADR-risk information for users of the mobile app. HCPs with smartphones will submit reports which capture details on patient socio-demographics, suspected medicine, other concurrent medicines, suspected ADR and medical history. The NPC transmits alerts via the mobile app on emerging medication-safety issues to users of the app.

Paper-form and web-form*:* The paper-form and web-form details will be distributed at all enrolled ART-sites. Copies of the paper-form are collected and forwarded to NPC for data capture in the national PV database and central analysis, prior to onward submission to the WHO database, VigiBase. HCPs also have the option to submit online ADR-reports using the web-form.

Economic cost data: Cost data will be collected by an appropriately trained RA. We will collect *setup costs* such as app development costs, equipment costs, cost of vehicles, training costs; and *running*

For peer review only - http://bmjopen.bmj.com/site/about/guidelines.xhtml

costs such as airtime, internet data, app maintenance, personnel costs, buildings and space if any, transport (fuel), stationary, brochures, costs of registers, and airtime/reminders. Both *setup* and *one-year running costs* will be collected from the provider perspective.

Feasibility and acceptability of the app*:* We will collect data on refusal rates and reasons for failed mobile app installation by consented HCPs in the intervention arm. We conducted a baseline qualitative study to assess the acceptability and barriers/facilitators of using the app by HCPs. We will conduct another qualitative evaluation after 1-year using 3-5 FGDs each with 6-8 HCPs and 20-30 in-depth interviews at 5% random ART-sites (~10) in the intervention arm.

Data Management

Training of research assistants*:* Both the initial and ongoing training focuses on the key concepts of PV, Good Clinical Practice, Human Subject Protection, randomization, risk profiles of ART and IPT; and techniques for collecting high-quality survey data; among others.

Data quality assurance*:* The PV database quality manager at NDA will be trained and empowered by the Research Team to embed data quality management in the national PV database.

Data protection*:* All ADR data are processed and securely stored by NPC. Paper-forms are manually entered into the PV database by authorized NPC staff while online data are electronically transmitted into VigiFlow and assessed before transfer into VigiBase. Researchers can request anonymized safety data for research purposes only. Paper-based questionnaires are stored under lock-and-key and accessed only by authorized project staff. Electronic data are password-protected and used for research purposes only. Data will be de-linked from HCP-identifiers prior to analysis.

Compliance with allocation: Prior to data anonymisation, an NPC officer not involved in the routine assessment of submitted ADR-reports will code these reports according to the allocated trial arm of the site where the report originates. Our team will use the codes to assess the extent of cross-over.

Data analysis

Success of randomization: Baseline characteristics (socio-demographics of HCPs, pattern of smartphone ownership by HCPs at ART-sites, geographical location of ART-site, nature & level of health facility, number & cadres of HCPs, number of PLHIV, and use of DTG and IPT) will be compared in both study arms to establish if randomization was successful.

Quantitative data: Analysis of results will be reported according to CONSORT guidelines and performed on both intention-to-treat and per protocol bases. All ADR-data received from the ~382 ART-sites during the study period will be downloaded from VigiBase and anonymized by the pharmacovigilance manager at NPC; and subsequently provided to the research team. Duplicate ADR-reports will be identified and excluded from formal statistical analyses. The randomization code will be broken after data analysis.

Unit of analysis: This unit will be an ADR-site in keeping with the cluster-randomized design.

Descriptive statistics: We shall determine the frequencies of de-duplicated HCP-reported ADRs per 100,000 treated-PLHIV per ART-site together with the time from ADR-onset to VigiBase registration.[26]

Effectiveness: Descriptive data will be aggregated to obtain the number of HCP-reported ADRs per 100,000 treated-PLHIV per trial arm and compared at cluster-level using the Student's t-test or Wilcoxon rank sum test, as appropriate, to determine if the app significantly increases ADR-reporting. We will assess if the introduction of Med Safety increases the rate of ADR-reporting by HCPs to NPC versus the use of existing PV methods (paper, online) alone during 30 months of follow-up and effect size of 25% at 5% level of statistical significance and power of 80%.

Multivariable analysis: Models will use the number of ADR-reports as the outcome. Hierarchical

For peer review only - http://bmjopen.bmj.com/site/about/guidelines.xhtml

models (level 1: ART-sites; level 2: HCPs nested within ART-sites; level 3: individuals nested within HCPs) will be fitted using maximum log-likelihood, considering intra-cluster correlations, using mixed models and generalized estimating equations models to control for potential confounders and effect modifiers identified at baseline assessment if fair randomization fails. Sub-group analyses will also be performed.

Feasibility and acceptability of the app*:* We will compute the refusal rates and detail the reasons for failed mobile app installation by consented HCPs in the intervention arm. Qualitative data on the acceptability and barriers/facilitators of using the mobile app by healthcare professionals will be analysed using thematic analysis by employing Nvivo 10 software.

Cost and cost-effectiveness: We will estimate unit *setup* and *running costs* for both the introduction of Med Safety in addition to existing PV methods and the use of existing PV methods alone. Costs per ADR-report submitted to NPC will be computed (overall; per study arm; per ADR attribute e.g. seriousness, avoidability, causality etc.). We will estimate cost-effectiveness of introducing Med Safety by calculating the Incremental Cost Effectiveness Ratio (ICER), which is the cost per additional ADR-report submitted to NPC.

Monitoring

Monitoring and Evaluation Mechanism: The Research Team will monitor the project's performance based on the research activities and report progress to the Project Steering Committee, which will sit (online) every six months. During monitoring, the Research Team will continually collect and analyse information on the project's ongoing research activities. The Steering Committee will provide independent external evaluation of the project based on the six-monthly reports from the Research Team.

Stopping rules: A Data Safety Monitoring Board is in place. Mid-term interim analysis will be conducted at $P < 0.01$ to assess if the app is substantially better than *a priori* estimates. The study will otherwise stop when all 382 ART-sites have been enrolled and followed up for 30-months. A protocol for patient care is in place at each ART-site to evaluate and manage PLHIV who develop suspected ADRs linked to ART and IPT.

Ethics and Dissemination

We obtained ethical approval for the study from the School of Biomedical Sciences Research and Ethics Committee at Makerere University College of Health Sciences (SBS-REC-720); and subsequently undertook research registration with the Uganda National Council for Science and Technology (HS1366ES). Administrative clearance is being obtained from participating ART-sites and written informed consent sought from participating HCPs, **supplementary file 3**.

Dissemination Strategies

During the study, limited communication tailored specifically for each study arm will be made by the study team to limit the effect of contamination.

Engaging Healthcare Providers**:** We shall employ video conferencing, webinars and face-to-face engagements to deliver continuing professional education sessions to HCPs on ADR-risk assessments for PLHIV prior to the initiation of DTG-regimens and IPT and the detection, management and reporting of ADRs for PLHIV already receiving DTG/IPT. Video conferencing is preferred to minimize the spread of COVID-19. If virtual meetings are not feasible, then face-to-face meetings will be held in full observance of the Ministry of Health's guidelines for limiting the spread of COVID-19: social distancing, use of face masks, and handwashing – especially for regional trainings of HCPs in remote areas where the internet might not be accessible and during the enrolment of study sites. The NPC in concert with Ministry of Health developed PV training materials for active drug safety monitoring and management of ADRs to DTG/IPT. We shall adapt and update

For peer review only - http://bmjopen.bmj.com/site/about/guidelines.xhtml

these materials with the results of our study for academic training and also tailor them for healthcare service delivery trainings to be conducted nationwide in partnership with NPC and Ministry of Health.

Reaching healthcare providers, Policymakers and Drug Regulators**:** This work underpins the wider rollout of Med Safety to improve pharmacovigilance across Uganda and beyond. This study is being implemented in direct partnership with ART-sites, Uganda's NDA which is the PV coordinating body in Uganda, UK's MHRA and WHO. Therefore, the study results will be readily available for immediate use to inform policy and practice by these key stakeholders. The NDA and MHRA (the software developers) will identify the barriers to the app's implementation and mitigate them as well as the facilitators and exploit them. Uganda's experience could be exploited to scale up the app in other low- and middle-income countries.

Reaching Researchers**:** In addition to social media and webinars, as above, we aim to reach researchers through traditional methods. We shall present our work at conferences including: Joint Annual Scientific Health Conference, Makerere University College of Health Sciences; International Society of Pharmacovigilance Annual Meeting; Conference on Retroviruses and Opportunistic Infections; International AIDS Society Conference, Royal Society of Tropical Medicine and Hygiene Annual Meeting; among others. We expect to produce at least six open access publications from this work, by the end of this study. We shall target journals including: Lancet Global Health; Drug Safety; Pharmacoepidemiology and Drug Safety, Journal of the International AIDS Society; Journal of Antimicrobial Chemotherapy; and Journal of Acquired Immune Deficiency Syndromes, etc.

Project Impact

Improving the reporting of suspected ADRs via digital PV is the first step towards boosting the volume of safety data available for robust signal detection analyses, improving understanding of the risk profiles of medicines and preventing future occurrence of avoidable ADRs. Improved PV translates into better recognition of serious ADRs and, ultimately, safer patient care. This work underpins future proposals to understand the risk factors for ADRs in PLHIV.

Protocol amendments

Protocol modifications that may impact on implementation of the study and affect patient safety including changes of study objectives, study design, study population, sample sizes, study procedures, or significant administrative aspects will require a formal protocol amendment. Such amendment will be agreed upon by the study investigators and approved by the School of Biomedical Sciences Research & Ethics Committee prior to implementation.

For peer review only

For peer review only - http://bmjopen.bmj.com/site/about/guidelines.xhtml

Patient and public involvement

Patients were not involved in designing this study and will not be directly involved in the conduct, reporting and dissemination of the research. However, healthcare professionals were involved in refining the study tools and will participate in conducting, reporting and disseminating the research.

Acknowledgements: UK's Medicines and Healthcare products Regulatory Agency and Uganda's National Drug Authority provided technical and logistical support during the planning and implementation of this project.

Contributors: RK, HBN, VB, CK, MP designed the study. RK drafted the manuscript. RK, NM, RS, HBN, VN, CK, KRK, PT, KH, CK, MP critically reviewed and revised the final version of the manuscript. All authors read and approved the final manuscript.

Funding: This project is supported by the Medical Research Council (MR/V03510X/1) and Makerere University Research & Innovations Fund (N/A).

Disclaimer: The funders had no role in study design, data collection and analysis, decision to publish or preparation of manuscripts.

Competing interests: None declared.

Patient consent for publication: Not required.

Provenance and peer review: Not commissioned; externally peer reviewed.

Open access: This is an open access article distributed in accordance with the Creative Commons Attribution Non-Commercial (CC BY-NC 4.0) license, which permits others to distribute, remix, adapt, build upon this work non-commercially, and license their derivative works on different terms, provided the original work is properly cited, appropriate credit is given, any changes made indicated, and the use is non-commercial. See: http:// creative commons. org/ licenses/ by- nc/ 4. 0/.

For peer review only - http://bmjopen.bmj.com/site/about/guidelines.xhtml

References

1. Grant AD, Mngadi KT, van Halsema CL, Luttig MM, Fielding KL, Churchyard GJ. Adverse events with isoniazid preventive therapy: experience from a large trial. *AIDS* 2010; **24**: S29-S36.
2. Kiguba R, Karamagi C, Waako P, Ndagije HB, Bird SM. Recognition and reporting of suspected adverse drug reactions by surveyed healthcare professionals in Uganda: key determinants. *BMJ Open* 2014; **4**(11): e005869.
3. Lepik KJ, Yip B, Ulloa AC, et al. Adverse drug reactions to integrase strand transfer inhibitors. *Aids* 2018; **32**(7): 903-12.
4. Pierce CE, de Vries ST, Bodin-Parssinen S, et al. Recommendations on the Use of Mobile Applications for the Collection and Communication of Pharmaceutical Product Safety Information: Lessons from IMI WEB-RADR. *Drug Saf* 2019; **42**(4): 477-89.
5. Bahk CY, Goshgarian M, Donahue K, et al. Increasing Patient Engagement in Pharmacovigilance Through Online Community Outreach and Mobile Reporting Applications: An Analysis of Adverse Event Reporting for the Essure Device in the US. *Pharmaceutical Medicine* 2015; **29**(6): 331-40.
6. Prakash J, Joshi K, Malik D, et al. "ADR PvPI" Android mobile app: Report adverse drug reaction at any time anywhere in India. *Indian J Pharmacol* 2019; **51**(4): 236-42.
7. Ministry of Health Uganda. Consolidated Guidelines for Prevention and Treatment of HIV in Uganda. 2018. http://library.health.go.ug/publications/hivaids/consolidated-guidelines-prevention-and-treatment-hiv-uganda (accessed 19 September 2019).
8. Ministry of Health Uganda. 100-Day Accelerated Isoniazid Preventive Therapy Scale Up Plan. 2019. https://health.go.ug/content/100-day-accelerated-isoniazid-preventive-therapy-scale-plan (accessed 19 September 2019).
9. Briggs MA, Emerson C, Modi S, Taylor NK, Date A. Use of Isoniazid Preventive Therapy for Tuberculosis, Prophylaxis among People Living with HIV/AIDS: A Review of the Literature. *J Acquir immune Defic Syndr* 2015; **68**.
10. GlaxoSmithKline. Prescribing Information: Dovato: GlaxoSmithKline, 2020.
11. Elzi L, Erb S, Furrer H, et al. Adverse events of raltegravir and dolutegravir. *Aids* 2017; **31**(13): 1853-8.
12. Hoffmann C, Welz T, Sabranski M, et al. Higher rates of neuropsychiatric adverse events leading to dolutegravir discontinuation in women and older patients. *HIV Med* 2017; **18**(1): 56-63.
13. Uganda. MoH. Consolidated guidelines for the prevention and treatment of HIV and AIDS in Uganda Kampala, Uganda: Ministry of Health Uganda, 2020.
14. Amuha MG, Kutyabami P, Kitutu FE, Odoi-Adome R, Kalyango JN. Non-adherence to anti-TB drugs among TB/HIV co-infected patients in Mbarara Hospital Uganda: prevalence and associated factors. *Afr Health Sci* 2009; **9 Suppl 1**(Suppl 1): S8-15.
15. Awofeso N. Anti-tuberculosis medication side-effects constitute major factor for poor adherence to tuberculosis treatment. *Bull World Health Organ* 2008; **86**(3): B-d.
16. Ghosh R, Lewis D. Aims and approaches of Web-RADR: a consortium ensuring reliable ADR reporting via mobile devices and new insights from social media. *Expert opinion on drug safety* 2015; **14**(12): 1845-53.
17. Oosterhuis I, Taavola H, Tregunno PM, et al. Characteristics, Quality and Contribution to Signal Detection of Spontaneous Reports of Adverse Drug Reactions Via the WEB-RADR Mobile Application: A Descriptive Cross-Sectional Study. *Drug Saf* 2018; **41**(10): 969-78.
18. Abadie D, Chebane L, Bert M, Durrieu G, Montastruc JL. Online reporting of adverse drug reactions: a study from a French regional pharmacovigilance center. *Therapie* 2014; **69**(5): 395-400.
19. Defer G, Le Caignec F, Fedrizzi S, et al. Dedicated mobile application for drug adverse reaction reporting by patients with relapsing remitting multiple sclerosis (Vigip-SEP study): study protocol for a randomized controlled trial. *Trials* 2018; **19**(1): 174.
20. Chan AW, Tetzlaff JM, Gotzsche PC, et al. SPIRIT 2013 explanation and elaboration: guidance for protocols of clinical trials. *BMJ* 2013; **346**: e7586.
21. Gallagher RM, Kirkham JJ, Mason JR, et al. Development and inter-rater reliability of the Liverpool adverse drug reaction causality assessment tool. *PLoS One* 2011; **6**(12): e28096.
22. Naranjo CA, Busto U, Sellers EM, et al. A method for estimating the probability of adverse drug reactions. *Clin Pharmacol Ther* 1981; **30**(2): 239-45.

For peer review only - http://bmjopen.bmj.com/site/about/guidelines.xhtml

23. Hayes RJ, Bennett S. Simple sample size calculation for cluster-randomized trials. *Int J Epidemiol* 1999; **28**(2): 319-26.
24. Ndagije HB, Kiguba R, Manirakiza L, et al. Healthcare professionals' perspective can guide post-marketing surveillance of artemisinin-based combination therapy in Uganda. *Malar J* 2020; **19**(1): 63.
25. Torgerson DJ. Contamination in trials: is cluster randomisation the answer? *Bmj* 2001; **322**(7282): 355-7.
26. Kiguba R, Ndagije HB, Nambasa V, Bird SM. Adverse Drug Reaction Onsets in Uganda's VigiBase(®): Delayed International Visibility, Data Quality and Illustrative Signal Detection Analyses. *Pharmaceut Med* 2018; **32**(6): 413-27.

For peer review only

For peer review only - http://bmjopen.bmj.com/site/about/guidelines.xhtml

Figures

Figure 1. Med Safety Mobile App

Figure 2. Flow diagram for cluster-randomized trial at full rollout

Supplementary Material

Supplementary file 1. Checklist for Standard Protocol Items: Recommendations for Interventional Trials 2013 statement

Supplementary file 2: Baseline questionnaires for healthcare professionals and facilities

Supplementary file 3: Informed Consent Form

For peer review only

For peer review only - http://bmjopen.bmj.com/site/about/guidelines.xhtml

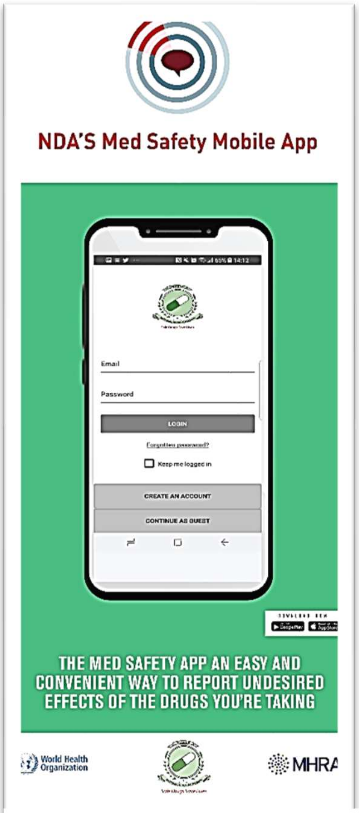

Figure 1: *Med Safety Mobile App*

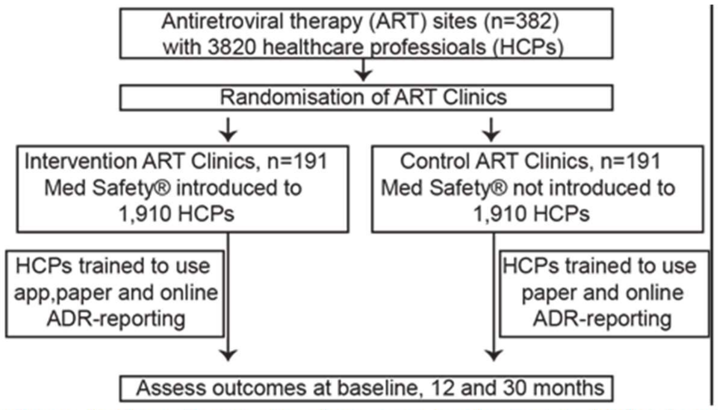

Figure 2. *Flow diagram for cluster-randomized trial at full roll-out*

Supplementary file 1. Checklist for Standard Protocol Items: Recommendations for Interventional Trials 2013 statement

		Reporting Item	Page and Line Number	Reason if not applicable
**Administrative information**				
Title	#1	Descriptive title identifying the study design, population, interventions, and, if applicable, trial acronym	Page 1, line 1	
Trial registration	#2a	Trial identifier and registry name. If not yet registered, name of intended registry	Page 2, line 26	
Trial registration: data set	#2b	All items from the World Health Organization Trial Registration Data Set	Yes (In trial register PACTR202009822379650)	
Protocol version	#3	Date and version identifier	N/A	Not used in the manuscript
Funding	#4	Sources and types of financial, material, and other support	Page 11, line 11	
Roles and responsibilities: contributorship	#5a	Names, affiliations, and roles of protocol contributors	Page 1, lines 6-29 and Page 11 , lines 8-10	
Roles and responsibilities: sponsor contact information	#5b	Name and contact information for the trial sponsor	N/A	
Roles and responsibilities: sponsor and funder	#5c	Role of study sponsor and funders, if any, in study design; collection, management, analysis, and interpretation of data; writing of the report;	Page 11, line 14	

For peer review only

		and the decision to submit the report for publication, including whether they will have ultimate authority over any of these activities		
Roles and responsibilities: committees	#5d	Composition, roles, and responsibilities of the coordinating centre, steering committee, endpoint adjudication committee, data management team, and other individuals or groups overseeing the trial, if applicable (see Item 21a for data monitoring committee)	Page 9, lines 25-29	
**Introduction**				
Background and rationale	#6a	Description of research question and justification for undertaking the trial, including summary of relevant studies (published and unpublished) examining benefits and harms for each intervention	Page 4, line 2 to Page 5, line 13	
Background and rationale: choice of comparators	#6b	Explanation for choice of comparators	Page 5, lines 11-13	
Objectives	#7	Specific objectives or hypotheses	Page 5, lines 16-26	
Trial design	#8	Description of trial design including type of trial (eg, parallel group, crossover, factorial, single group), allocation ratio, and framework (eg, superiority, equivalence, non-inferiority, exploratory)	Page 5, line 21	
**Methods: Participants, interventions, and outcomes**				

For peer review only

Study setting	#9	Description of study settings (eg, community clinic, academic hospital) and list of countries where data will be collected. Reference to where list of study sites can be obtained	Page 5, line 29	
Eligibility criteria	#10	Inclusion and exclusion criteria for participants. If applicable, eligibility criteria for study centres and individuals who will perform the interventions (eg, surgeons, psychotherapists)	Page 5, line 34	
Interventions: description	#11a	Interventions for each group with sufficient detail to allow replication, including how and when they will be administered	Page 5, line 38	
Interventions: modifications	#11b	Criteria for discontinuing or modifying allocated interventions for a given trial participant (eg, drug dose change in response to harms, participant request, or improving / worsening disease)	N/A	No discontinuations/modifications are planned. Both intention-to-treat and per protocol analyses will be conducted
Interventions: adherance	#11c	Strategies to improve adherence to intervention protocols, and any procedures for monitoring adherence (eg, drug tablet return; laboratory tests)	Page 5, lines 41-42 and Page 8, lines 26-28	
Interventions: concomitant care	#11d	Relevant concomitant care and interventions that are permitted or prohibited during the trial	Page 9, line 38	
Outcomes	#12	Primary, secondary, and other outcomes, including the specific measurement variable (eg, systolic blood pressure), analysis metric (eg, change from baseline, final value, time to event),	Page 5, line 52 to Page 6 line 5	

For peer review only

		method of aggregation (eg, median, proportion), and time point for each outcome. Explanation of the clinical relevance of chosen efficacy and harm outcomes is strongly recommended		
Participant timeline	#13	Time schedule of enrolment, interventions (including any run-ins and washouts), assessments, and visits for participants. A schematic diagram is highly recommended (see Figure)	Page 6, line 7-12	
Sample size	#14	Estimated number of participants needed to achieve study objectives and how it was determined, including clinical and statistical assumptions supporting any sample size calculations	Page 6, line 14	
Recruitment	#15	Strategies for achieving adequate participant enrolment to reach target sample size	Page 7, lines 2-13	
**Methods: Assignment of interventions (for controlled trials)**				
Allocation: sequence generation	#16a	Method of generating the allocation sequence (eg, computer-generated random numbers), and list of any factors for stratification. To reduce predictability of a random sequence, details of any planned restriction (eg, blocking) should be provided in a separate document that is unavailable to those who enrol participants or assign interventions	Page 7, line 21	

For peer review only

Allocation concealment mechanism	#16b	Mechanism of implementing the allocation sequence (eg, central telephone; sequentially numbered, opaque, sealed envelopes), describing any steps to conceal the sequence until interventions are assigned	Page 7, line 26	
Allocation: implementation	#16c	Who will generate the allocation sequence, who will enrol participants, and who will assign participants to interventions	Page 7, line 39	
Blinding (masking)	#17a	Who will be blinded after assignment to interventions (eg, trial participants, care providers, outcome assessors, data analysts), and how	Page 7, line 21-22	
Blinding (masking): emergency unblinding	#17b	If blinded, circumstances under which unblinding is permissible, and procedure for revealing a participant's allocated intervention during the trial	N/A	
**Methods: Data collection, management, and analysis**				
Data collection plan	#18a	Plans for assessment and collection of outcome, baseline, and other trial data, including any related processes to promote data quality (eg, duplicate measurements, training of assessors) and a description of study instruments (eg, questionnaires, laboratory tests) along with their reliability and validity, if known. Reference to where data collection forms can be found, if not in the protocol	Page 7, line 35 to Page 8, line 9, supplementary file 2	

For peer review only

Data collection plan: retention	#18b	Plans to promote participant retention and complete follow-up, including list of any outcome data to be collected for participants who discontinue or deviate from intervention protocols	N/A	This is a pragmatic trial in which no other interruption to real-life practice will be made – apart from introducing the mobile application at baseline and sending monthly mobile phone short message reminders for 6-months.
Data management	#19	Plans for data entry, coding, security, and storage, including any related processes to promote data quality (eg, double data entry; range checks for data values). Reference to where details of data management procedures can be found, if not in the protocol	Page 8, line 12-28	
Statistics: outcomes	#20a	Statistical methods for analysing primary and secondary outcomes. Reference to where other details of the statistical analysis plan can be found, if not in the protocol	Page 8, line 31 to Page 9, line 5	
Statistics: additional analyses	#20b	Methods for any additional analyses (eg, subgroup and adjusted analyses)	Page 9, lines 4	
Statistics: analysis population and missing data	#20c	Definition of analysis population relating to protocol non-adherence (eg, as randomised analysis), and any statistical methods to handle missing data (eg, multiple imputation)	Page 8, line 38	
**Methods: Monitoring**				
Data monitoring: formal committee	#21a	Composition of data monitoring committee (DMC); summary of its role and reporting structure; statement of whether it is	Page 9, line 25	

For peer review only

		independent from the sponsor and competing interests; and reference to where further details about its charter can be found, if not in the protocol. Alternatively, an explanation of why a DMC is not needed		
Data monitoring: interim analysis	#21b	Description of any interim analyses and stopping guidelines, including who will have access to these interim results and make the final decision to terminate the trial	Page 9, line 26	
Harms	#22	Plans for collecting, assessing, reporting, and managing solicited and spontaneously reported adverse events and other unintended effects of trial interventions or trial conduct	N/A	Introduction of the Med Safety App in the intervention arm is thought to be of very minimal risk.
Auditing	#23	Frequency and procedures for auditing trial conduct, if any, and whether the process will be independent from investigators and the sponsor	Page 9, line 26	
**Ethics and dissemination**				
Research ethics approval	#24	Plans for seeking research ethics committee / institutional review board (REC / IRB) approval	Page 9, line 32]	
Protocol amendments	#25	Plans for communicating important protocol modifications (eg, changes to eligibility criteria, outcomes, analyses) to relevant parties (eg, investigators, REC / IRBs, trial participants, trial registries, journals, regulators)	Page 10, line 27	

For peer review only

Consent or assent	#26a	Who will obtain informed consent or assent from potential trial participants or authorised surrogates, and how (see Item 32)	Page 9, line 36	
Consent or assent: ancillary studies	#26b	Additional consent provisions for collection and use of participant data and biological specimens in ancillary studies, if applicable	N/A	No biological samples will be collected.
Confidentiality	#27	How personal information about potential and enrolled participants will be collected, shared, and maintained in order to protect confidentiality before, during, and after the trial	Page 7, line 19-24	
Declaration of interests	#28	Financial and other competing interests for principal investigators for the overall trial and each study site	Page 11, line 16	
Data access	#29	Statement of who will have access to the final trial dataset, and disclosure of contractual agreements that limit such access for investigators	Page 11, line 16	
Ancillary and post trial care	#30	Provisions, if any, for ancillary and post-trial care, and for compensation to those who suffer harm from trial participation	N/A	Introduction of the Med Safety App is thought to have very minimal risk for study participants.
Dissemination policy: trial results	#31a	Plans for investigators and sponsor to communicate trial results to participants, healthcare professionals, the public, and other relevant groups (eg, via publication, reporting in results databases, or other data sharing	Page 9 lines 38 to Page 10, line 19	

For peer review only

		arrangements), including any publication restrictions		
Dissemination policy: authorship	#31b	Authorship eligibility guidelines and any intended use of professional writers	Page 11, line 8-10	
Dissemination policy: reproducible research	#31c	Plans, if any, for granting public access to the full protocol, participant-level dataset, and statistical code	Page 11, line 19-23	
**Appendices**				
Informed consent materials	#32	Model consent form and other related documentation given to participants and authorised surrogates	Supplementary file 3	
Biological specimens	#33	Plans for collection, laboratory evaluation, and storage of biological specimens for genetic or molecular analysis in the current trial and for future use in ancillary studies, if applicable	N/A	Biological specimens will not be collected

It is strongly recommended that this checklist be read in conjunction with the SPIRIT 2013 Explanation & Elaboration for important clarification on the items. Amendments to the protocol should be tracked and dated. The SPIRIT checklist is copyrighted by the SPIRIT Group under the Creative Commons "Attribution-NonCommercial-NoDerivs 3.0 Unported" license.

For peer review only

Study Questionnaire for Healthcare Professionals

Research Assistant: ____________________ Code: [ ]

Region: ____________________ Code: [ ]

Health Facility Name: ____________________ Code: [ ]

Date of Interview: ____/_____/_____

Adverse drug reaction (ADR) is an unwanted or harmful reaction experienced following the administration of a drug or combination of drugs under normal doses/conditions of use & is suspected to be related to the drug. An ADR might require the drug to be discontinued or the dose reduced.

SECTION A: DEMOGRAPHICS

1. Gender:
   [1] Male
   [2] Female
2. Age (*in completed years*): _____
3. Do you own a working smartphone?
   [1] Yes
   [2] No

   3a) If ***YES*** to **Q3**, are you willing to join a WhatsApp group? [1] Yes [2] No

   3b) If ***YES*** to **Q3a**, can you share your WhatsApp number? ____________
4. Do you own a simple working mobile phone *(not a smartphone)*?
   [1] Yes
   [2] No
5. Highest Education Level:
   [1] Certificate
   [2] Diploma
   [3] Bachelors
   [4] Masters
   [5] Other, (*specify*)____________
6. Professional experience (*Years*): [ ]
7. If less than 1 year in **Q5**, state number of *completed months* _____
8. Do you use any of the following? (***Tick all that apply***)
   [1] WhatsApp
   [2] Facebook
   [3] Twitter
   [4] Other, (*specify*) ____________
9. Professional Cadre:
   [1] Physician
   [2] Medical Officer
   [3] Pharmacist
   [4] Nurse/Midwife
   [5] Clinical Officer
   [6] Pharmacy Technician
   [7] Lay Counsellor
   [8] Expert Client
   [9] Other, (*specify*) ____________
10. Health Facility Type:
    [1] Public
    [2] Private Not-for-Profit
    [3] Private for-Profit
11. Health Facility Status:
    [1] Regional Referral Hospital
    [2] Hospital
    [3] Health Centre IV
    [4] Health Centre III
    [5] Health Centre II
    [6] Private Clinic
    [7] Other, (*specify*) ____________

For peer review only - http://bmjopen.bmj.com/site/about/guidelines.xhtml

SECTION B: DOLUTEGRAVIR-LINKED AND ISONIAZID-LINKED ADRS

Please, complete the questionnaire by indicating the appropriate responses.

1. What is the approximate number of HIV-positive patients you see per day? _____

Dolutegravir-linked adverse drug reactions

2. Dolutegravir-based antiretroviral therapy was recently rolled out in the treatment of HIV-positive patients, have you ***ever encountered*** any dolutegravir-linked adverse drug reactions (ADRs) amongst the HIV-positive patients in your care?
   [1] Yes [2] No [9] I don't know

3. In the past 4-weeks, have you ***suspected*** any dolutegravir-linked ADRs?
   [1] Yes [2] No (***Skip to 5***) [9] I don't know (***Skip to 5***)

4. If ***YES*** to **Q3**, how many cases of dolutegravir-linked ADRs have you ***suspected*** in the past 4-weeks? _____

5. In the past 4-weeks, have you ***received*** any ***patient-complaints*** of dolutegravir-linked ADRs?
   [1] Yes [2] No (***Skip to 7***) [9] I don't know (***Skip to 7***)

6. If ***YES*** to **Q5**, how many ***patient-complaints*** of dolutegravir-linked ADRs have you ***received*** in the past 4-weeks? _______

7. Briefly describe the most recent ***dolutegravir-linked*** ADR you have encountered giving details on ***patient age***, ***sex***, ***approximate ADR-date, details of the adverse drug reaction***, ***severity*** *(mild, moderate, severe)*, ***seriousness*** (*led to or prolonged hospitalization, caused incapacitation, led to any other medically significant condition or death*), ***clinical outcome*** & ***action taken***:

   Patient age [ ] years; **sex** [M or F]; **approximate ADR-date** [*mm/yyyy*: __ / ____]

   ADR description (*include severity, seriousness, clinical outcome and action taken*)____
   ____
   ____
   ____
   ____
   ____

Isoniazid preventive therapy-linked adverse drug reactions

8. HIV-positive patients are at a higher risk of dying when they develop active tuberculosis (TB). During the past one year, Ministry of Health scaled up the roll-out of isoniazid preventive therapy (IPT) among HIV-positive patients to significantly reduce these patients' risk of developing active TB. IPT, however, has been associated with ADRs. Have you ***ever encountered*** any IPT-linked ADRs amongst the HIV-positive patients in your care?
   [1] Yes [2] No [9] I don't know

9. In the past 4-weeks, have you ***suspected*** any isoniazid preventive therapy-linked ADRs?
   [1] Yes [2] No (***Skip to 11***) [9] I don't know (***Skip to 11***)

For peer review only - http://bmjopen.bmj.com/site/about/guidelines.xhtml

10. If ***YES*** to **Q9**, how many isoniazid preventive therapy-linked ADRs have you ***suspected*** in the *past 4-weeks*? _____

11. In the past 4-weeks**,** have you ***received*** any ***patient-complaints*** of isoniazid preventive therapy-linked ADRs?

    [1] Yes [2] No (***Skip to 13***) [9] I don't know (***Skip to 13***)

12. If ***YES*** to **Q11**, how many ***patient-complaints*** of isoniazid preventive therapy-linked ADRs have you ***received*** in the past 4-weeks? _____

13. Briefly describe the most recent ***isoniazid-linked*** ADR you have encountered giving details on ***patient age***, ***sex***, ***approximate ADR-date, details of the adverse drug reaction***, ***severity*** *(mild, moderate, severe)*, ***seriousness*** (*led to or prolonged hospitalization, caused incapacitation, led to any other medically significant condition or death*), ***clinical outcome*** & ***action taken***:

    Patient age [ ] years; **sex** [M or F]; **approximate ADR-date** [*mm/yyyy*: __ / ____]

    ADR description (*include severity, seriousness, clinical outcome and action taken*)____
    ____
    ____
    ____
    ____
    ____

14. In the past 6-months, have you ***reported*** any suspected ADRs experienced by the HIV-positive patients under your care?

    [1] Yes [2] No (***Skip to Q23***) [9] I don't know (***Skip to Q23***)

15. If ***YES*** to **Q14**, how many ***ADR reports*** did you make? [ ][ ]

16. If ***YES*** to **Q14**, which drug classes were suspected? (***Tick all that apply***)

    [1] Antiretroviral therapy

    [2] Isoniazid preventive therapy

    [3] Antituberculosis therapy

    [4] Other, (*specify*)____

17. If ***YES*** to **Q14**, which of the following drugs were implicated? (***Tick all that apply and mention number of reported ADRs in past 4-weeks for options [1] & [2]***)

    [1] Dolutegravir: *Number of ADRs* ***reported*** *in the past 4-weeks* _____

    [2] Isoniazid: *Number of ADRs* ***reported*** *in the past 4-weeks* _____

    [3] Other, (*specify*) ______________

For peer review only - http://bmjopen.bmj.com/site/about/guidelines.xhtml

18. If ***YES*** to **Q14**, to whom did you report the most recent ADR? (***Tick all that apply***)
    [1] District Health Officer
    [2] Health Management Information System
    [3] Immediate Supervisor
    [4] National Drug Authority - National Pharmacovigilance Center
    [5] Other, (*specify*)__________________

19. If ***YES*** to **Q14**, how did you report the most recent suspected ADR? (***Tick all that apply***)
    [1] Verbally
    [2] Written paper report
    [3] Written online/website report
    [4] Other, (*specify*) __________________

20. If ***YES*** to **Q14**, did you get feedback on the ADR(s) you reported?
    [1] Yes [2] No [9] Not sure

21. If ***YES*** to **Q20**, what form of feedback?
    ____
    ____

22. In special situations, the National Drug Authority may need additional information about reported ADRs. Would you be willing to answer further questions after sending a report?
    [1] Yes [2] No [9] I don't know

Herbal Medicine Use

23. In the past 6-months, have you suspected that some of your patients use herbal medicines together with ARVs? [1] Yes [2] No

24. If YES to **Q23**, have you inquired from the patients whether they use herbal medicines together with ARVs? [1] Yes [2] No

25. In the past six months, have any of your patients reported to you that they use herbal medicines together with ARVs? [1] Yes [2] No

26. Have any of your patients presented with ADRs likely to be linked to the use of herbal medicines? [1] Yes [2] No

We appreciate the time you've taken to respond to this survey. Thank you!

For peer review only - http://bmjopen.bmj.com/site/about/guidelines.xhtml

Health Facility Data

Research Assistant: ______________________ Code: [ ]

Region: _________________________________ Code: [ ]

Health Facility Name: _______________________________ Code: [ ]

Date of Enrolment of Health Facility: ____/_____/_____

Average daily number of ART patient visits at the Health Facility: [ ][ ][ ]

Total number of patients on dolutegravir-containing antiretroviral therapy [ ][ ][ ][ ]

Total Number of patients on Isoniazid Preventive Therapy [ ][ ][ ][ ]

Number of healthcare professionals at the Health Facility:

- Medical Doctors [ ][ ]
- Pharmacists [ ][ ]
- Clinical Officers [ ][ ]
- Nurses/Midwives [ ][ ]
- Pharmacy Technicians [ ][ ]
- Village Health Teams [ ][ ]
- Lay Counsellors [ ][ ]
- Expert Clients [ ][ ]
- Other (*specify*) ……………………….. [ ][ ]

Does the Health Facility provide health workers with internet access?
1. No 2. Yes 9. Unknown

If YES, describe the form of internet access provided (*wireless, wired, data etc.*):

____

____

Is mobile telephone network connectivity available at the Health Facility?
1. No 2. Yes 9. Unknown

If YES, describe the reliability of mobile telephone network connectivity:

____

____

For peer review only - http://bmjopen.bmj.com/site/about/guidelines.xhtml

Informed Consent Form

Purpose of the study: Experts from National Drug Authority and Makerere University College of Health Sciences are working together to improve the rate and quality of reporting of adverse drug reactions linked to the newly rolled-out dolutegravir-containing antiretroviral therapy and scaled up isoniazid preventive therapy to reduce the risk of tuberculosis in HIV-positive people. This project focuses on the involvement of healthcare professionals in the active drug safety monitoring and management of adverse drug reactions to these drugs and how this system can be improved.

Procedures: In this survey, we ask you to recall and describe the most recent suspected and/or reported adverse drug reactions (ADRs) linked to the use of dolutegravir-containing antiretroviral therapy and isoniazid preventive therapy among the HIV-positive people in your care; and any ADRs you have suspected in the past 4-weeks and/or ADR-complaints you have received from HIV patients in the past 4-weeks. The survey is anonymous and will take no more than 20-30 minutes to complete. After the interview, we shall provide you with the tools you will use to report any adverse drug reactions you encounter, particularly those linked to the use of dolutegravir and isoniazid preventive therapy. You will be contacted by phone at the end of the study to obtain feedback from you about your experience while using the tools for reporting adverse drug reactions. You could even be invited to participate in a group or individual in-depth discussion to share your experiences.

Time compensation: We greatly appreciate your time and opinion. The time you spend filling in this questionnaire will be compensated at a rate of UGX 20,000.

Confidentiality: Any information obtained in this study that can identify you will remain confidential and be disclosed only with your permission or as required by law. Otherwise, confidentiality will be maintained because the self-completed questionnaire is anonymous. We will not request for your name in the information we get from this study.

Participation and withdrawal: Participation is voluntary and you can withdraw at any time.

Identification of Investigators: If you have any questions or concerns about the research, please feel free to contact: Dr. Ronald Kiguba, Principal Investigator, Department of Pharmacology & Therapeutics, School of Biomedical Sciences, Makerere University College of Health Sciences, Tel: +256712840683 & email: kiguba@yahoo.co.uk

Rights of participants: The School of Biomedical Sciences Research & Ethics Committee and the Uganda National Council for Science & Technology (UNCST) have reviewed my request to conduct this project. If you have any concerns about your rights in this study, please contact Dr. Erisa Mwaka Tel: +256 752575050 & email: erisamwaka@gmail.com OR UNCST on +256 414 705500 / +256 312 314800

I understand the procedures described above. My questions have been answered to my satisfaction, and I agree to participate in this study. I have been given a copy of this form.

Signature of participant: ……………………………… Date: ……………………………………

Signature of research assistant: ……………………… Date: ……………………………………

For peer review only

BMJ Open

Effectiveness of the Med Safety mobile application in improving adverse drug reaction reporting by healthcare professionals in Uganda: a protocol for a pragmatic cluster-randomized controlled trial

Journal:	*BMJ Open*
Manuscript ID	bmjopen-2022-061725.R1
Article Type:	Protocol
Date Submitted by the Author:	08-Jun-2022

Complete List of Authors:	Kiguba, Ronald; Makerere University College of Health Sciences, Pharmacology and Therapeutics<br>Mwebaza, Norah; Makerere University College of Health Sciences, Pharmacology and Therapeutics<br>Ssenyonga, Ronald ; Makerere University, Epidemiology & Biostatistics<br>Ndagije, Helen ; National Drug Authority, National Pharmacovigilance Centre<br>Nambasa, Victoria; National Drug Authority, National Pharmacovigilance Centre<br>Katureebe, Cordelia; Ministry of Health<br>Katumba, Kenneth; MRC/UVRI and LSHTM Uganda Research Unit<br>Tregunno, Phil; Medicines and Healthcare Products Regulatory Agency<br>Harrison, Kendal; Medicines and Healthcare Products Regulatory Agency<br>Karamagi, Charles; Makerere University College of Health Sciences, Clinical Epidemiology Unit, Department of Medicine<br>Scott, Kathryn A.; University of Liverpool, MRC Centre for Drug Safety Science and Wolfson Centre for Personalised Medicine, Institute of Systems, Molecular and Integrative Biology (ISMIB)<br>Pirmohamed, Munir; University of Liverpool, MRC Centre for Drug Safety Science and Wolfson Centre for Personalised Medicine, Institute of Systems, Molecular and Integrative Biology (ISMIB)

**Primary Subject Heading**:	Pharmacology and therapeutics
Secondary Subject Heading:	Epidemiology, Health economics, HIV/AIDS, Public health, Qualitative research
Keywords:	Adverse events < THERAPEUTICS, CLINICAL PHARMACOLOGY, EPIDEMIOLOGY, Epidemiology < INFECTIOUS DISEASES

SCHOLARONE™
Manuscripts

Effectiveness of the Med Safety mobile application in improving adverse drug reaction reporting by healthcare professionals in Uganda: a protocol for a pragmatic cluster-randomized controlled trial

Authors:

Ronald Kiguba[1], Norah Mwebaza[1], Ronald Ssenyonga[2], Helen B. Ndagije[3], Victoria Nambasa[3], Cordelia Katureebe[4], Kenneth R. Katumba[5], Phil Tregunno[6], Kendal Harrison[6], Charles Karamagi[7], Kathryn A. Scott[8], Munir Pirmohamed[8]*

*Correspondence to: Ronald Kiguba (Tel: +256 701 840683; Email: kiguba@gmail.com)

Affiliations:

[1]Department of Pharmacology and Therapeutics, College of Health Sciences, Makerere University, Kampala, Uganda

[2]Department of Epidemiology and Biostatistics, College of Health Sciences, Makerere University, Kampala, Uganda

[3]National Pharmacovigilance Centre, National Drug Authority, Kampala, Uganda

[4]AIDS Control Programme, Ministry of Health, Kampala, Uganda

[5]MRC/UVRI & LSHTM Uganda Research Unit

[6]Medicines and Healthcare products Regulatory Agency, London, United Kingdom

[7]Clinical Epidemiology Unit, College of Health Sciences, Makerere University, Kampala, Uganda

[8]MRC Centre for Drug Safety Science and Wolfson Centre for Personalised Medicine, Institute of Systems, Molecular and Integrative Biology (ISMIB), University of Liverpool, Liverpool, United Kingdom

For peer review only

For peer review only - http://bmjopen.bmj.com/site/about/guidelines.xhtml

Abstract

Introduction: Combination antiretroviral therapy (cART) has massively reduced HIV mortality. However, long-term cART increases the risk of adverse drug reactions (ADRs), which can lead to higher morbidity, mortality, and healthcare costs for people living with HIV (PLHIV).

Pharmacovigilance – monitoring the effects of medicines – is essential for understanding real-world drug safety. In Uganda, pharmacovigilance systems have only recently been developed and rates of ADR-reporting for cART are very low. Thus, the safety profile of medicines currently used to treat HIV and tuberculosis in our population is poorly understood.

The Med Safety mobile application has been developed through the European Union's Innovative Medicines Initiative WEB-Recognising Adverse Drug Reactions project to promote digital pharmacovigilance. This mobile application has been approved for ADR-reporting by Uganda's National Drug Authority. However, the barriers and facilitators to Med Safety uptake, and its effectiveness in improving pharmacovigilance are as yet unknown.

Methods and analysis: A pragmatic cluster-randomized controlled trial will be implemented over 30-months at 191 intervention and 191 comparison cART-sites to evaluate Med Safety. Using a randomization sequence generated by the sealed envelope software, we shall randomly assign the 382 pre-screened cART-sites to the intervention and comparison arms. Each cART-site is a cluster which consists of healthcare professionals and PLHIV receiving dolutegravir-based cART and/or Isoniazid Preventive Therapy. Healthcare professionals enrolled in the intervention arm will be trained in the use of mobile-, paper- and web-based reporting, whilst those in the comparison arm will be trained in paper- and web-based reporting only.

Ethics and dissemination: Ethical approval was given by the School of Biomedical Sciences Research and Ethics Committee at Makerere University (SBS-REC-720) and administrative clearance obtained from Uganda National Council for Science and Technology (HS1366ES). Study results will be shared with healthcare professionals, policy-makers, the public and academia.

Registration: PACTR202009822379650.

For peer review only

For peer review only - http://bmjopen.bmj.com/site/about/guidelines.xhtml

Strengths and limitations of the study

- This study will be delivered by researchers, policymakers, software developers, healthcare providers and consumers to promote medication safety in resource-limited settings.
- We aim to recruit 3,820 healthcare professionals from 382 combination antiretroviral therapy sites spread across Uganda; the selected study sites serve 80% of people living with HIV receiving combination antiretroviral therapy.
- A limitation is that the use of the Med Safety mobile app requires a smartphone but only 16% of Ugandans own a smartphone; limited rollout showed that 7 in 10 healthcare professionals are smartphone owners.

For peer review only

For peer review only - http://bmjopen.bmj.com/site/about/guidelines.xhtml

Introduction

Spontaneous adverse drug reaction (ADR)-reporting is the backbone of pharmacovigilance (PV) globally but is plagued by under-reporting.[1-3] Only 3% of Ugandan healthcare professionals (HCPs) reported a suspected ADR to the National Pharmacovigilance Centre (NPC) in 2014.[2] These HCPs cited lack of conventional paper-based ADR-forms (paper-forms), limited access to web-based forms (web-forms) due to the scarcity of internet-wired computers, and the lack of feedback to ADR-reporters as common barriers to PV. Also, logistical challenges lurk in the countrywide distribution and collection of paper-forms by Uganda's NPC, which hinders timely data collation and analysis.[2] Ultimately, the implementation of requisite policy actions to safeguard the public from medication-related harm has been delayed.

Innovative interventions are needed to improve ADR-reporting, such as the use of mobile applications (mobile apps or apps) to support traditional PV.[4] Mobile apps have had variable success in PV worldwide. A 10-fold increase in the rate of ADR-reporting was seen for a medical device with social media engagement in the USA. In contrast, uptake of mobile app reporting has been poor in Europe: studies in the United Kingdom, the Netherlands and Croatia showed 1.7, 1.1 and 6.5 app-ADR-reports per completed-month per 1,000 app-downloads, respectively.[4, 5] Uptake was also poor in India: 4.0 app-ADR-reports per completed-month per 1,000 app-downloads.[6]

Efforts to strengthen PV in Uganda are timely due to the recent rollout of dolutegravir (DTG) as the preferred drug for first-line and second-line combination antiretroviral therapy (cART). DTG is more effective and tolerable, and has a higher genetic barrier to developing drug resistance than other antiretrovirals.[7] Rollout began in October 2018 and, by August 2019, over 347,888 PLHIV were receiving DTG-based cART.[7] In addition, Uganda is rapidly scaling up Isoniazid Preventive Therapy (IPT) for the prevention of tuberculosis (TB) in people living with HIV (PLHIV). Only 16% of PLHIV had been reached with life-saving IPT by June 2019:[8] an extra 135,711 PLHIV were initiated on IPT by September 2019 after a 100-day scale-up campaign.[7, 8] IPT significantly reduces the incidence of active TB, curbing TB-related mortality among PLHIV.[9]

Although DTG is generally well tolerated, it is associated with serious ADRs including hyperglycaemia,[10-13] neuropsychiatric effects (1.7%)[14] and hepatotoxicity (0.1%).[15] DTG is also associated with more common, but less serious, ADRs such as headache, abnormal dreams and abdominal pain, but the impact of these has not been evaluated in real-world settings in developing countries. The rapid rollout of DTG and scale up of IPT will increase the number of PLHIV exposed to these therapies, potentially increasing the incidence of ADRs, including serious ADRs, associated with these medicines. Discontinuation of DTG and/or IPT is recommended if serious ADRs such as jaundice, blurred vision or hyperglycaemia occur.[16] Four in 100,000 PLHIV who receive IPT die due to an IPT-related adverse event.[1]

Adverse drug reactions can reduce quality of life for PLHIV, and increase morbidity, mortality, and healthcare costs.[17, 18] In 2016, prior to DTG rollout, the NPC attempted to improve spontaneous ADR-reporting via a web-form. However, they received only 92 online reports versus 290 paper-based ADR-reports linked to cART from October 2018 to September 2019. More recently, a PV task-force comprised of the NPC, the AIDS Control Program and Ministry of Health (MoH) was set up. The task-force established an active drug safety monitoring and management program to complement the spontaneous ADR-reporting system. The NPC received 109 DTG-linked ADR-reports from October 2018 to September 2019 from ~348,000 PLHIV. Only 18 IPT-linked ADR-reports were received from January 2019 to June 2019 from ~300,000 PLHIV: 2.6 DTG-linked ADR-reports per month per 100,000 treated-PLHIV and 1.0 IPT-linked ADR-report per month per 100,000 treated-PLHIV. These rates are barely 5% the known incidence of the ADRs in PLHIV elsewhere.[1, 3]

The Med Safety mobile application was developed by the European Union Innovative Medicines Initiative: WEB-Recognising Adverse Drug Reactions (WEB-RADR). The same technology that produced the Med Safety App was also adopted by European countries including UK's Medicines and Healthcare products Regulatory Agency (MHRA) in July 2015, Netherlands' Lareb in January 2016 and Croatia's HALMED in May 2016. In 2017 the Med Safety App was introduced in Africa (Burkina Faso, Zambia) in partnership with World Health Organization (WHO). MHRA adapted Med Safety for low and middle-income countries (LMICs) including Uganda with approval from Uganda's National Drug Authority (NDA), **Figure 1**.[4],[19] Med Safety was launched in limited settings in Uganda in February 2020.

For peer review only - http://bmjopen.bmj.com/site/about/guidelines.xhtml

Med Safety's platform facilitates easy adoption and low-cost maintenance in LMICs. It is available at no cost for mobile phones and tablets, for Android and iOS, in English. Within the app, ADR-reports can be completed offline and transmitted to NPC when internet connectivity is established. The ADR-reporting form has a clear and simple format. App-users can browse and view ADR-data and may create a "watch-list" of *medicines* of their own interest to receive personalized news and alerts. Med Safety provides for two-way exchange of medication-safety information between NPC and HCPs. This enhanced interaction promotes the involvement of HCPs in PV activities. The anticipated increase in volume of ADR-reports in the national PV database could enhance its signal detection potential,[20] and contribute to remedial efforts to improve patient safety. The app is also integrated into the WHO Collaborating Centre for International Drug Monitoring – Uppsala Monitoring Centre (WHO-UMC) application program interfaces (including Vigiflow, VigiAccess, WHODrug).[19] This study seeks to understand whether Med Safety is effective in improving ADR-reporting by HCPs if used together with traditional PV methods versus if traditional PV methods are used alone.

Aims and objectives

Our aim is to assess the feasibility, effectiveness, cost and cost-effectiveness of implementing Med Safety for HCP-driven reporting of ADRs associated with DTG-containing cART and IPT for TB prevention in PLHIV in Uganda. Our hypothesis is that the use of Med Safety for ADR-reporting by HCPs attending to PLHIV on DTG-based cART and/or IPT will increase the ADR-reporting-rate[21, 22] by at least 25% versus use of existing PV methods alone in 30 months of follow-up at selected cART-sites in Uganda. We will test our hypothesis in a pragmatic multi-centre open-label cluster-randomized controlled trial whose specific objectives are to:

1. Assess the feasibility and acceptability of Med Safety for ADR-reporting by HCPs at selected cART-sites in Uganda
2. Determine the app's effect on the rate of ADR-reporting versus traditional PV methods alone
3. Estimate the app's cost and cost-effectiveness from the provider perspective

Methods and Analysis

Participants, interventions and outcomes

Study setting: The study will be conducted nationwide at 382 (of 1,832 cART-sites) high volume accredited cART-sites in Uganda. The 382 cART-sites serve 80% of the PLHIV on cART in Uganda.[8] This protocol follows the Standard Protocol Items: Recommendations for Interventional Trials 2013 statement, **supplementary file 1**.[23]

Eligibility criteria: All smartphone-owning HCPs at these sites are eligible including: physicians, medical officers, pharmacists, nurses and midwives, clinical officers, pharmacy technicians and community health workers (lay counsellors and expert clients). Limited rollout showed that 7 in 10 HCPs are smartphone owners. Written informed consent will be sought from eligible HCPs. We expect to enrol 10 HCPs per cART-site on average.

Intervention arm: HCPs at intervention cART-sites will be introduced to the Med Safety mobile app whether they own a smartphone or not. Mobile app, paper-form and web-form awareness campaigns including initial face-to-face training, posters/brochures and monthly reminder WhatsApp and mobile-phone short messages (SMS) for up to 6-months will be undertaken. Training will be conducted by pharmacists from NPC and Makerere University with expertise in PV. The training teams have harmonized the training schedule to ensure uniform training. Interested HCPs with personal smartphones will be invited and assisted to install the mobile app and trained to use it to report suspected ADRs, with emphasis on DTG- and IPT-linked ADRs. HCPs will also be trained and encouraged to use traditional PV methods (paper-form, web-form).

Comparison arm: HCPs at the comparison cART-sites will be trained and encouraged to use traditional methods of ADR-reporting (paper-form, web-form). All aspects of the training will be

For peer review only - http://bmjopen.bmj.com/site/about/guidelines.xhtml

identical to those in the intervention arm except that Med Safety will not be introduced to HCPs in the comparison arm. Reminder WhatsApp and SMS about the paper-form and web-form will be sent out monthly for up to 6-months.

Outcomes*:* Our primary outcome is number of HCP-reported ADRs per 100,000 person-months of treated-PLHIV per study arm. Our secondary outcomes are number of app-ADR-reports per 1,000 app-downloads per month of follow-up; causality (by Naranjo Scale and Liverpool Causality Assessment Tool)[24, 25]; seriousness as per the WHO definitions (threatens life i.e. leads to or prolongs hospitalization, causes incapacitation or death); ADR-outcome; cost per ADR-report; cost per additional ADR-report, cost per additional avoidable serious ADR-report.

Participant timeline: There are 15 regional referral hospitals each with a catchment of ~25 cART-sites. Each team of three research assistants requires four weeks to enrol HCPs in one regional catchment. Follow-up at each cART-site begins after its enrolment and lasts 30 months. The 30-month follow-up period is considered adequate to monitor durability of the real-life impact of the app on ADR-reporting. Four additional months will be required to wrap-up the study, thus, 36-months overall, **Table 1**.

Table 1. Schedule of enrolment, interventions and assessments.

	STUDY PERIOD						
	**Baseline**	**Follow-up schedule**					**Close-out**
**Procedures**	**(t_0)**	**t_0+3 months**	**t_0+6 months**	**t_0+12 months**	**t_0+15 months**	**t_0+30 months**	**t_0+36 months**
**ENROLMENT:**							
Eligibility screen	X						
Informed consent	X						
Allocation to study arm	X						
**INTERVENTIONS:**							
Mobile App Arm	X	X	X	X	X	X	
Comparison Arm	X	X	X	X	X	X	
**ASSESSMENTS:**							
Quantitative Survey	X			X			
Mobile App Data	X	X	X	X	X	X	
Economic Cost Data	X	X	X	X			
Interim Data Analysis					X		
Qualitative Study	X			X			
Endline Data Analysis							X

For peer review only

For peer review only - http://bmjopen.bmj.com/site/about/guidelines.xhtml

Sample Size: To estimate the number of cART-sites required per arm for an effect size of 25%:[26] we assume power of 80% at the 95% confidence level, mean of 1.0 IPT-linked ADR/month/100,000 treated-PLHIV (0.00001) and standard deviation (SD) of 1.179 IPT-linked ADRs/month/100,000 treated-PLHIV (0.00001179) (SD was computed based on the monthly IPT-linked ADR-reports submitted to NPC for one-year from October 2018 to September 2019), cluster-size of 10 HCPs and coefficient of variation of 0.25,[26] which yields ~114 cART-sites per trial-arm or 228 in both study arms. A minimum of 189 cART-sites per arm or 378 in both study arms (66% increment) will be required to cater for the following limitations; i) the large proportion of HCPs who do not own smartphones of ~50-60% as previously estimated[21, 22] – and is 26% [95% confidence interval (CI) of 23% to 30%] from limited rollout of RCT, ii) refusal to participate by eligible HCPs of ~5% as previously estimated[27] – and is 0% [95% CI of 0% to 2%] from limited rollout of RCT, iii) loss to follow-up due to the relatively long follow-up period of 30-months (~10%) and, iv) contamination due to information sharing between study arms through social media (~30%).[28] We shall enrol 382 cART-sites (191 per study arm) to cater for other unforeseen limitations; thus, recruiting up to 3,820 HCPs (1,910 HCPs per trial-arm). Limited rollout of the RCT suggests a cluster size of 7.2 (629/87) HCPs. We shall retain the average cluster size of 10 HCPs because the RCT has not yet enrolled from the central region of Uganda (with the country's capital city) where cART-sites tend to be much larger with more HCPs per site than the more rural eastern, western and northern regions.

Assignment of interventions

Unit of randomization: This unit is a cluster, defined as an cART-site located at each of the 382 pre-screened health facilities and consisting of HCPs and DTG/IPT-treated PLHIV. The use of cART-sites as clusters minimizes co-intervention, duplication of reporting, and organizational challenges, and provides valid PLHIV population denominators for analysis of the outcomes.

Concealment of sequence generation*:* Using the sealed envelope software, an independent Biostatistician from the Clinical Epidemiology Unit at Makerere University, who will not participate in the subsequent data analyses, generated the block randomization sequence to ensure balanced allocation of clusters.

Randomization: The pre-screened cART-sites will be assigned by research assistants to the intervention and comparison arms, **Figure 2**, using an electronically generated block randomization sequence.

Blinding: PLHIV, HCPs, PV assessors of the ADR-reports submitted to NPC and the Biostatistician who will analyze the data will be blinded to the allocation of cART-sites. The research assistants will not be blinded to the allocation of cART-sites due to the nature of the intervention.

Data collection

Baseline survey: Consented HCPs will complete an interviewer-administered electronic questionnaire using the Open Data Kit (ODK) software to record participants' characteristics and details on ADR-reporting. Details of the questionnaires are provided in **supplementary file 2**.

Mobile app data: The app is hosted by NDA where the NPC is located, which is also the preferred source of ADR-risk information for users of the mobile app. HCPs with smartphones will submit reports which capture details on patient socio-demographics, suspected medicine, other concurrent medicines, suspected ADR and medical history. The NPC transmits alerts via the mobile app on emerging medication-safety issues to users of the app.

Paper-form and web-form*:* The paper-form and web-form details will be distributed at all enrolled cART-sites. Pharmacovigilance focal persons based at the cART-sites and NDA regulatory officers will routinely collect the paper-forms and forward them to NPC for data capture in the national PV database and central analysis, prior to onward submission to the WHO database, VigiBase. HCPs also have the option to submit online ADR-reports using the web-form.

For peer review only - http://bmjopen.bmj.com/site/about/guidelines.xhtml

Economic cost data: Cost data will be collected by an appropriately trained RA. We will collect *setup costs* such as app development costs, equipment costs, cost of vehicles, training costs; and *running costs* such as airtime, internet data, app maintenance, personnel costs, buildings and space if any, transport (fuel), stationary, brochures, costs of registers, and airtime/reminders. Both *setup* and *one-year running costs* will be collected from the provider perspective.

Feasibility and acceptability of the app: Prior to trial implementation, we will conduct a baseline qualitative study to gauge the acceptability and feasibility of introducing the app to HCPs. We will conduct 3-5 Focus Group Discussions each with 5-8 HCPs and 20-30 in-depth interviews in a random 6% of cART-sites (~12) in the intervention arm. During trial implementation, we will document the refusal and failure rates to install the app among consented HCPs in the intervention arm. App-users will be asked to report their experiences to gauge app-feasibility, assess user-satisfaction, and identify potential revisions to the app. We will gauge acceptability of the app based on whether users can recommend the app to other HCPs to report suspected ADRs to NPC.

Data Management

Training of research assistants*:* Both the initial and ongoing training focuses on the key concepts of PV, Good Clinical Practice, Human Subject Protection, randomization, risk profiles of cART and IPT; and techniques for collecting high-quality survey data; among others.

Data quality assurance*:* The PV database quality manager at NDA will be trained and empowered by the Research Team to embed data quality management in the national PV database.

Data protection*:* All ADR data are processed and securely stored by NPC. Paper-forms are manually entered into the PV database by authorized NPC staff while online data are electronically transmitted into VigiFlow and assessed before transfer into VigiBase. Researchers can request anonymized safety data for research purposes only. Paper-based questionnaires are stored under lock-and-key and accessed only by authorized project staff. Electronic data are password-protected and used for research purposes only. Data will be de-linked from HCP-identifiers prior to analysis.

Compliance with allocation: Prior to data anonymisation, an NPC officer not involved in the routine assessment of submitted ADR-reports will code these reports according to the allocated trial arm of the site where the report originates. Our team will use the codes to assess the extent of cross-over.

Data analysis

Success of randomization: Baseline characteristics (socio-demographics of HCPs, pattern of smartphone ownership by HCPs at cART-sites, geographical location of cART-site, nature & level of health facility, number & cadres of HCPs, number of PLHIV, and use of DTG and IPT) will be compared in both study arms to establish if randomization was successful.

Quantitative data: Analysis of results will be reported according to CONSORT guidelines and performed on both intention-to-treat and per protocol bases. All ADR-data received from the ~382 cART-sites during the study period will be downloaded from VigiBase and anonymized by the pharmacovigilance manager at NPC; and subsequently provided to the research team. Duplicate ADR-reports will be identified and excluded from formal statistical analyses. The randomization code will be broken after data analysis.

Unit of analysis: This unit will be an ADR-site in keeping with the cluster-randomized design.

Analysis of missing or lost clusters: We will compare baseline characteristics of individuals in the lost clusters with the characteristics of individuals in the clusters that will have completed follow-up. If we find no significant differences between the clusters, we will conclude that our results don't include any differential misclassification. If there are differences, however, we will report this finding and discuss its implications.

Descriptive statistics: We shall determine the frequencies of de-duplicated HCP-reported ADRs per

For peer review only - http://bmjopen.bmj.com/site/about/guidelines.xhtml

100,000 treated-PLHIV per cART-site together with the time from ADR-onset to VigiBase registration.[29]

Effectiveness: Descriptive data will be aggregated to obtain the number of HCP-reported ADRs per 100,000 treated-PLHIV per trial arm and compared at cluster-level using the Student's t-test or Wilcoxon rank sum test, as appropriate, to determine if the app significantly increases ADR-reporting. We will assess if the introduction of Med Safety increases the rate of ADR-reporting by HCPs to NPC versus the use of existing PV methods (paper, online) alone during 30 months of follow-up and effect size of 25% at 5% level of statistical significance and power of 80%.

Multivariable analysis: Models will use the number of ADR-reports as the outcome. Hierarchical models (level 1: cART-sites; level 2: HCPs nested within cART-sites; level 3: individuals nested within HCPs) will be fitted using maximum log-likelihood, considering intra-cluster correlations, using mixed models and generalized estimating equations models to control for potential confounders and effect modifiers identified at baseline assessment if fair randomization fails. Sub-group analyses will also be performed.

Feasibility and acceptability of the app*:* We will compute the refusal rates and detail the reasons for failed mobile app installation by consented HCPs in the intervention arm. Qualitative data on the acceptability and barriers/facilitators of using the mobile app by HCPs will be analysed using thematic analysis by employing Nvivo 10 software.

Cost and cost-effectiveness: We will estimate unit *setup* and *running costs* for both the introduction of Med Safety in addition to existing PV methods and the use of existing PV methods alone. Costs per ADR-report submitted to NPC will be computed (overall; per study arm; per ADR attribute e.g. seriousness, avoidability, causality etc.). We will estimate cost-effectiveness of introducing Med Safety by calculating the Incremental Cost Effectiveness Ratio (ICER), which is the cost per additional ADR-report submitted to NPC.

Monitoring

Monitoring and Evaluation Mechanism: The Research Team will monitor the project's performance based on the research activities and report progress to the Project Steering Committee, which will sit (online) every six months. During monitoring, the Research Team will continually collect and analyse information on the project's ongoing research activities. The Steering Committee will provide independent external evaluation of the project based on the six-monthly reports from the Research Team.

Stopping rules: A Data Safety Monitoring Board is in place. Mid-term interim analysis will be conducted at $P < 0.01$ to assess if the app is substantially better than *a priori* estimates. The study will otherwise stop when all 382 cART-sites have been enrolled and followed up for 30-months. A protocol for patient care is in place at each cART-site to evaluate and manage PLHIV who develop suspected ADRs linked to cART and IPT.

Ethics and Dissemination

We obtained ethical approval for the study from the School of Biomedical Sciences Research and Ethics Committee at Makerere University College of Health Sciences (SBS-REC-720); and subsequently undertook research registration with the Uganda National Council for Science and Technology (HS1366ES). Administrative clearance is being obtained from participating cART-sites and written informed consent sought from participating HCPs, **supplementary file 3**.

Dissemination Strategies

During the study, limited communication tailored specifically for each study arm will be made by the study team to limit the effect of contamination.

For peer review only - http://bmjopen.bmj.com/site/about/guidelines.xhtml

Engaging Healthcare Providers: We shall employ video conferencing, webinars and face-to-face engagements to deliver continuing professional education sessions to HCPs on ADR-risk assessments for PLHIV prior to the initiation of DTG-regimens and IPT and the detection, management and reporting of ADRs for PLHIV already receiving DTG/IPT. Video conferencing is preferred to minimize the spread of COVID-19. If virtual meetings are not feasible, then face-to-face meetings will be held in full observance of the Ministry of Health's guidelines for limiting the spread of COVID-19: social distancing, use of face masks, and handwashing – especially for regional trainings of HCPs in remote areas where the internet might not be accessible and during the enrolment of study sites. The NPC in concert with Ministry of Health developed PV training materials for active drug safety monitoring and management of ADRs to DTG/IPT. We shall adapt and update these materials with the results of our study for academic training and also tailor them for healthcare service delivery trainings to be conducted nationwide in partnership with NPC and Ministry of Health.

Reaching healthcare providers, Policymakers and Drug Regulators: This work underpins the wider rollout of Med Safety to improve pharmacovigilance across Uganda and beyond. This study is being implemented in direct partnership with cART-sites, Uganda's NDA which is the PV coordinating body in Uganda, UK's MHRA and WHO. Therefore, the study results will be readily available for immediate use to inform policy and practice by these key stakeholders. The NDA and MHRA (the software developers) will identify the barriers to the app's implementation and mitigate them as well as the facilitators and exploit them. Uganda's experience could be exploited to scale up the app in other low- and middle-income countries.

Reaching Researchers: In addition to social media and webinars, as above, we aim to reach researchers through traditional methods. We shall present our work at conferences including: Joint Annual Scientific Health Conference, Makerere University College of Health Sciences; International Society of Pharmacovigilance Annual Meeting; Conference on Retroviruses and Opportunistic Infections; International AIDS Society Conference, Royal Society of Tropical Medicine and Hygiene Annual Meeting; among others. We expect to produce at least six open access publications from this work, by the end of this study. We shall target journals including: Lancet Global Health; Drug Safety; Pharmacoepidemiology and Drug Safety, Journal of the International AIDS Society; Journal of Antimicrobial Chemotherapy; and Journal of Acquired Immune Deficiency Syndromes, etc.

Project Impact

Improving the reporting of suspected ADRs via digital PV is the first step towards boosting the volume of safety data available for robust signal detection analyses, improving understanding of the risk profiles of medicines and preventing future occurrence of avoidable ADRs. Improved PV translates into better recognition of serious ADRs and, ultimately, safer patient care. This work underpins future proposals to understand the risk factors for ADRs in PLHIV.

Protocol amendments

Protocol modifications that may impact on implementation of the study and affect patient safety including changes of study objectives, study design, study population, sample sizes, study procedures, or significant administrative aspects will require a formal protocol amendment. Such amendment will be agreed upon by the study investigators and approved by the School of Biomedical Sciences Research & Ethics Committee prior to implementation.

For peer review only - http://bmjopen.bmj.com/site/about/guidelines.xhtml

Patient and public involvement

Patients were not involved in designing this study and will not be directly involved in the conduct, reporting and dissemination of the research. However, HCPs were involved in refining the study tools and will participate in conducting, reporting and disseminating the research.

Acknowledgements: UK's Medicines and Healthcare products Regulatory Agency and Uganda's National Drug Authority provided technical and logistical support during the planning and implementation of this project.

Contributors: RK, HBN, VB, CK, MP designed the study. RK drafted the manuscript. RK, NM, RS, HBN, VN, CK, KRK, PT, KH, CK, MP critically reviewed and revised the final version of the manuscript. All authors read and approved the final manuscript.

Funding: This project is supported by the Medical Research Council (MR/V03510X/1) and Makerere University Research & Innovations Fund (N/A).

Disclaimer: The funders had no role in study design, data collection and analysis, decision to publish or preparation of manuscripts.

Competing interests: None declared.

Patient consent for publication: Not required.

Provenance and peer review: Not commissioned; externally peer reviewed.

Open access: This is an open access article distributed in accordance with the Creative Commons Attribution Non-Commercial (CC BY-NC 4.0) license, which permits others to distribute, remix, adapt, build upon this work non-commercially, and license their derivative works on different terms, provided the original work is properly cited, appropriate credit is given, any changes made indicated, and the use is non-commercial. See: http:// creative commons. org/ licenses/ by- nc/ 4. 0/.

For peer review only

References

1. Grant, A.D., et al., *Adverse events with isoniazid preventive therapy: experience from a large trial.* AIDS, 2010. **24**: p. S29-S36.
2. Kiguba, R., et al., *Recognition and reporting of suspected adverse drug reactions by surveyed healthcare professionals in Uganda: key determinants.* BMJ Open, 2014. **4**(11): p. e005869.
3. Lepik, K.J., et al., *Adverse drug reactions to integrase strand transfer inhibitors.* Aids, 2018. **32**(7): p. 903-912.
4. Pierce, C.E., et al., *Recommendations on the Use of Mobile Applications for the Collection and Communication of Pharmaceutical Product Safety Information: Lessons from IMI WEB-RADR.* Drug Saf, 2019. **42**(4): p. 477-489.
5. Bahk, C.Y., et al., *Increasing Patient Engagement in Pharmacovigilance Through Online Community Outreach and Mobile Reporting Applications: An Analysis of Adverse Event Reporting for the Essure Device in the US.* Pharmaceutical Medicine, 2015. **29**(6): p. 331-340.
6. Prakash, J., et al., *"ADR PvPI" Android mobile app: Report adverse drug reaction at any time anywhere in India.* Indian J Pharmacol, 2019. **51**(4): p. 236-242.
7. Ministry of Health Uganda. *Consolidated Guidelines for Prevention and Treatment of HIV in Uganda*. 2018 [cited 2019 19 September]; Available from: http://library.health.go.ug/publications/hivaids/consolidated-guidelines-prevention-and-treatment-hiv-uganda.
8. Ministry of Health Uganda. *100-Day Accelerated Isoniazid Preventive Therapy Scale Up Plan*. 2019 [cited 2019 19 September]; Available from: https://health.go.ug/content/100-day-accelerated-isoniazid-preventive-therapy-scale-plan.
9. Briggs, M.A., et al., *Use of Isoniazid Preventive Therapy for Tuberculosis, Prophylaxis among People Living with HIV/AIDS: A Review of the Literature.* J Acquir immune Defic Syndr, 2015. **68**.
10. GlaxoSmithKline, *Prescribing Information: Dovato*. 2020, GlaxoSmithKline.
11. Cassenote, A.J.F., et al., *Incidence and associated factors of type 2 diabetes mellitus onset in the Brazilian HIV/AIDS cohort study.* Braz J Infect Dis, 2021. **25**(4): p. 101608.
12. Lamorde, M., et al., *Dolutegravir-associated hyperglycaemia in patients with HIV.* Lancet HIV, 2020. **7**(7): p. e461-e462.
13. O'Halloran, J.A., et al., *Integrase Strand Transfer Inhibitors are Associated with Incident Diabetes Mellitus in People with HIV.* Clin Infect Dis, 2022.
14. Elzi, L., et al., *Adverse events of raltegravir and dolutegravir.* Aids, 2017. **31**(13): p. 1853-1858.
15. Hoffmann, C., et al., *Higher rates of neuropsychiatric adverse events leading to dolutegravir discontinuation in women and older patients.* HIV Med, 2017. **18**(1): p. 56-63.
16. Uganda., M.o.H., *Consolidated guidelines for the prevention and treatment of HIV and AIDS in Uganda Kampala, Uganda*. 2020, Ministry of Health Uganda.
17. Amuha, M.G., et al., *Non-adherence to anti-TB drugs among TB/HIV co-infected patients in Mbarara Hospital Uganda: prevalence and associated factors.* Afr Health Sci, 2009. **9 Suppl 1**(Suppl 1): p. S8-15.
18. Awofeso, N., *Anti-tuberculosis medication side-effects constitute major factor for poor adherence to tuberculosis treatment.* Bull World Health Organ, 2008. **86**(3): p. B-d.
19. Ghosh, R. and D. Lewis, *Aims and approaches of Web-RADR: a consortium ensuring reliable ADR reporting via mobile devices and new insights from social media.* Expert Opin Drug Saf, 2015. **14**(12): p. 1845-53.
20. Oosterhuis, I., et al., *Characteristics, Quality and Contribution to Signal Detection of Spontaneous Reports of Adverse Drug Reactions Via the WEB-RADR Mobile Application: A Descriptive Cross-Sectional Study.* Drug Saf, 2018. **41**(10): p. 969-978.
21. Abadie, D., et al., *Online reporting of adverse drug reactions: a study from a French regional pharmacovigilance center.* Therapie, 2014. **69**(5): p. 395-400.
22. Defer, G., et al., *Dedicated mobile application for drug adverse reaction reporting by patients with relapsing remitting multiple sclerosis (Vigip-SEP study): study protocol for a randomized controlled trial.* Trials, 2018. **19**(1): p. 174.

For peer review only - http://bmjopen.bmj.com/site/about/guidelines.xhtml

23. Chan, A.W., et al., *SPIRIT 2013 explanation and elaboration: guidance for protocols of clinical trials.* BMJ, 2013. **346**: p. e7586.
24. Gallagher, R.M., et al., *Development and inter-rater reliability of the Liverpool adverse drug reaction causality assessment tool.* PLoS One, 2011. **6**(12): p. e28096.
25. Naranjo, C.A., et al., *A method for estimating the probability of adverse drug reactions.* Clin Pharmacol Ther, 1981. **30**(2): p. 239-45.
26. Hayes, R.J. and S. Bennett, *Simple sample size calculation for cluster-randomized trials.* Int J Epidemiol, 1999. **28**(2): p. 319-326.
27. Ndagije, H.B., et al., *Healthcare professionals' perspective can guide post-marketing surveillance of artemisinin-based combination therapy in Uganda.* Malar J, 2020. **19**(1): p. 63.
28. Torgerson, D.J., *Contamination in trials: is cluster randomisation the answer?* Bmj, 2001. **322**(7282): p. 355-7.
29. Kiguba, R., et al., *Adverse Drug Reaction Onsets in Uganda's VigiBase(®): Delayed International Visibility, Data Quality and Illustrative Signal Detection Analyses.* Pharmaceut Med, 2018. **32**(6): p. 413-427.

For peer review only

For peer review only - http://bmjopen.bmj.com/site/about/guidelines.xhtml

Figures

Figure 1. Med Safety Mobile App

Figure 2. Flow diagram for cluster-randomized trial at full rollout

Supplementary Material

Supplementary file 1. Checklist for Standard Protocol Items: Recommendations for Interventional Trials 2013 statement

Supplementary file 2: Baseline questionnaires for healthcare professionals and facilities

Supplementary file 3: Informed Consent Form

For peer review only

For peer review only - http://bmjopen.bmj.com/site/about/guidelines.xhtml

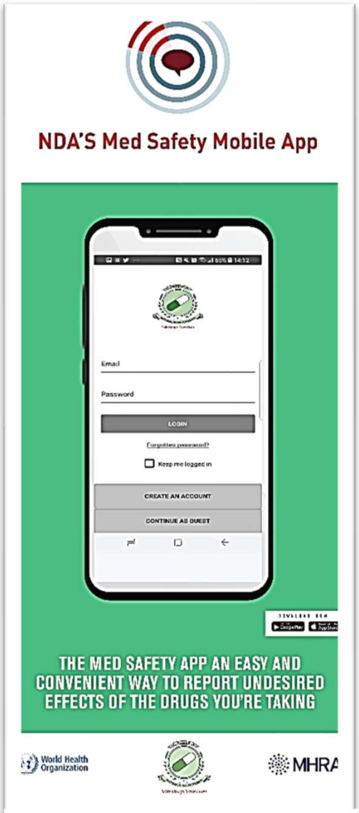

Figure 1: *Med Safety Mobile App*

For peer review only

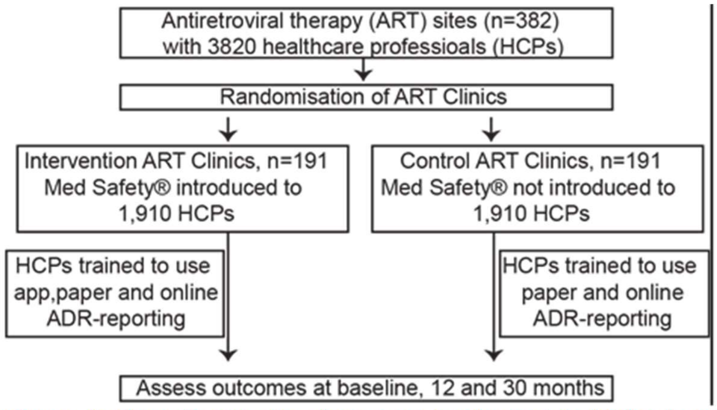

Figure 2. *Flow diagram for cluster-randomized trial at full roll-out*

Supplementary file 1. Checklist for Standard Protocol Items: Recommendations for Interventional Trials 2013 statement

		Reporting Item	Page and Line Number	Reason if not applicable
**Administrative information**				
Title	#1	Descriptive title identifying the study design, population, interventions, and, if applicable, trial acronym	Page 1, line 1	
Trial registration	#2a	Trial identifier and registry name. If not yet registered, name of intended registry	Page 2, line 26	
Trial registration: data set	#2b	All items from the World Health Organization Trial Registration Data Set	Yes (In trial register PACTR202009822379650)	
Protocol version	#3	Date and version identifier	N/A	Not used in the manuscript
Funding	#4	Sources and types of financial, material, and other support	Page 11, line 11	
Roles and responsibilities: contributorship	#5a	Names, affiliations, and roles of protocol contributors	Page 1, lines 6-29 and Page 11 , lines 8-10	
Roles and responsibilities: sponsor contact information	#5b	Name and contact information for the trial sponsor	N/A	
Roles and responsibilities: sponsor and funder	#5c	Role of study sponsor and funders, if any, in study design; collection, management, analysis, and interpretation of data; writing of the report; and the decision to submit the report for	Page 11, lines 13-14	

For peer review only

		publication, including whether they will have ultimate authority over any of these activities		
Roles and responsibilities: committees	#5d	Composition, roles, and responsibilities of the coordinating centre, steering committee, endpoint adjudication committee, data management team, and other individuals or groups overseeing the trial, if applicable (see Item 21a for data monitoring committee)	Page 9, lines 28-33	
**Introduction**				
Background and rationale	#6a	Description of research question and justification for undertaking the trial, including summary of relevant studies (published and unpublished) examining benefits and harms for each intervention	Page 4, line 2 to Page 5, line 13	
Background and rationale: choice of comparators	#6b	Explanation for choice of comparators	Page 5, lines 11-13	
Objectives	#7	Specific objectives or hypotheses	Page 5, lines 16-26	
Trial design	#8	Description of trial design including type of trial (eg, parallel group, crossover, factorial, single group), allocation ratio, and framework (eg, superiority, equivalence, non-inferiority, exploratory)	Page 5, line 21-22	
**Methods: Participants, interventions, and outcomes**				
Study setting	#9	Description of study settings (eg, community clinic, academic hospital) and list of countries	Page 5, line 29-32	

For peer review only

		where data will be collected. Reference to where list of study sites can be obtained		
Eligibility criteria	#10	Inclusion and exclusion criteria for participants. If applicable, eligibility criteria for study centres and individuals who will perform the interventions (eg, surgeons, psychotherapists)	Page 5, line 34-38	
Interventions: description	#11a	Interventions for each group with sufficient detail to allow replication, including how and when they will be administered	Page 5, line 40-48	
Interventions: modifications	#11b	Criteria for discontinuing or modifying allocated interventions for a given trial participant (eg, drug dose change in response to harms, participant request, or improving / worsening disease)	N/A	No discontinuations/modifications are planned. Both intention-to-treat and per protocol analyses will be conducted
Interventions: adherence	#11c	Strategies to improve adherence to intervention protocols, and any procedures for monitoring adherence (eg, drug tablet return; laboratory tests)	Page 5, lines 43-44 and Page 8, lines 30-32	
Interventions: concomitant care	#11d	Relevant concomitant care and interventions that are permitted or prohibited during the trial	Page 9, line 47-48	
Outcomes	#12	Primary, secondary, and other outcomes, including the specific measurement variable (eg, systolic blood pressure), analysis metric (eg, change from baseline, final value, time to event), method of aggregation (eg, median, proportion), and time point for each outcome. Explanation of	Page 6, lines 3-8	

For peer review only

		the clinical relevance of chosen efficacy and harm outcomes is strongly recommended		
Participant timeline	#13	Time schedule of enrolment, interventions (including any run-ins and washouts), assessments, and visits for participants. A schematic diagram is highly recommended (see Figure)	Page 6, line 10-15	
Sample size	#14	Estimated number of participants needed to achieve study objectives and how it was determined, including clinical and statistical assumptions supporting any sample size calculations	Page 7, lines 1-18	
Recruitment	#15	Strategies for achieving adequate participant enrolment to reach target sample size	Page 7, lines 7-15	
**Methods: Assignment of interventions (for controlled trials)**				
Allocation: sequence generation	#16a	Method of generating the allocation sequence (eg, computer-generated random numbers), and list of any factors for stratification. To reduce predictability of a random sequence, details of any planned restriction (eg, blocking) should be provided in a separate document that is unavailable to those who enrol participants or assign interventions	Page 7, line 26-29	
Allocation concealment mechanism	#16b	Mechanism of implementing the allocation sequence (eg, central telephone; sequentially numbered, opaque, sealed envelopes),	Page 7, line 28	

For peer review only

		describing any steps to conceal the sequence until interventions are assigned		
Allocation: implementation	#16c	Who will generate the allocation sequence, who will enrol participants, and who will assign participants to interventions	Page 7, line 31-33	
Blinding (masking)	#17a	Who will be blinded after assignment to interventions (eg, trial participants, care providers, outcome assessors, data analysts), and how	Page 7, line 35-37	
Blinding (masking): emergency unblinding	#17b	If blinded, circumstances under which unblinding is permissible, and procedure for revealing a participant's allocated intervention during the trial	N/A	
**Methods: Data collection, management, and analysis**				
Data collection plan	#18a	Plans for assessment and collection of outcome, baseline, and other trial data, including any related processes to promote data quality (eg, duplicate measurements, training of assessors) and a description of study instruments (eg, questionnaires, laboratory tests) along with their reliability and validity, if known. Reference to where data collection forms can be found, if not in the protocol	Page 7, line 40 to Page 8, line 13, supplementary file 2	
Data collection plan: retention	#18b	Plans to promote participant retention and complete follow-up, including list of any outcome data to be collected for participants who	N/A	This is a pragmatic trial in which no other interruption to real-life practice will be made – apart from introducing the mobile application at baseline and

For peer review only

		discontinue or deviate from intervention protocols		sending monthly mobile phone short message reminders for 6-months.
Data management	#19	Plans for data entry, coding, security, and storage, including any related processes to promote data quality (eg, double data entry; range checks for data values). Reference to where details of data management procedures can be found, if not in the protocol	Page 8, line 16-32	
Statistics: outcomes	#20a	Statistical methods for analysing primary and secondary outcomes. Reference to where other details of the statistical analysis plan can be found, if not in the protocol	Page 8, line 35 to Page 9, line 26	
Statistics: additional analyses	#20b	Methods for any additional analyses (eg, subgroup and adjusted analyses)	Page 9, lines 13-14	
Statistics: analysis population and missing data	#20c	Definition of analysis population relating to protocol non-adherence (eg, as randomised analysis), and any statistical methods to handle missing data (eg, multiple imputation)	Page 8, lines 41, 48	
**Methods: Monitoring**				
Data monitoring: formal committee	#21a	Composition of data monitoring committee (DMC); summary of its role and reporting structure; statement of whether it is independent from the sponsor and competing interests; and reference to where further details about its charter can be found, if not in the protocol. Alternatively, an explanation of why a DMC is not needed	Page 9, line 34	

For peer review only

Data monitoring: interim analysis	#21b	Description of any interim analyses and stopping guidelines, including who will have access to these interim results and make the final decision to terminate the trial	Page 9, line 35	
Harms	#22	Plans for collecting, assessing, reporting, and managing solicited and spontaneously reported adverse events and other unintended effects of trial interventions or trial conduct	N/A	Introduction of the Med Safety App in the intervention arm is thought to be of very minimal risk.
Auditing	#23	Frequency and procedures for auditing trial conduct, if any, and whether the process will be independent from investigators and the sponsor	Page 9, line 35	
**Ethics and dissemination**				
Research ethics approval	#24	Plans for seeking research ethics committee / institutional review board (REC / IRB) approval	Page 9, line 41-44]	
Protocol amendments	#25	Plans for communicating important protocol modifications (eg, changes to eligibility criteria, outcomes, analyses) to relevant parties (eg, investigators, REC / IRBs, trial participants, trial registries, journals, regulators)	Page 10, line 37-41	
Consent or assent	#26a	Who will obtain informed consent or assent from potential trial participants or authorised surrogates, and how (see Item 32)	Page 9, line 45	
Consent or assent: ancillary studies	#26b	Additional consent provisions for collection and use of participant data and biological specimens in ancillary studies, if applicable	N/A	No biological samples will be collected.

For peer review only

Confidentiality	#27	How personal information about potential and enrolled participants will be collected, shared, and maintained in order to protect confidentiality before, during, and after the trial	Page 7, line 23-28	
Declaration of interests	#28	Financial and other competing interests for principal investigators for the overall trial and each study site	Page 11, line 15	
Data access	#29	Statement of who will have access to the final trial dataset, and disclosure of contractual agreements that limit such access for investigators	Page 11, line 18	
Ancillary and post trial care	#30	Provisions, if any, for ancillary and post-trial care, and for compensation to those who suffer harm from trial participation	N/A	Introduction of the Med Safety App is thought to have very minimal risk for study participants.
Dissemination policy: trial results	#31a	Plans for investigators and sponsor to communicate trial results to participants, healthcare professionals, the public, and other relevant groups (eg, via publication, reporting in results databases, or other data sharing arrangements), including any publication restrictions	Page 9 lines 47 to Page 10, line 29	
Dissemination policy: authorship	#31b	Authorship eligibility guidelines and any intended use of professional writers	Page 11, line 8-10	
Dissemination policy: reproducible research	#31c	Plans, if any, for granting public access to the full protocol, participant-level dataset, and statistical code	Page 11, line 18-22	

For peer review only

Appendices				
Informed consent materials	#32	Model consent form and other related documentation given to participants and authorised surrogates	Supplementary file 3	
Biological specimens	#33	Plans for collection, laboratory evaluation, and storage of biological specimens for genetic or molecular analysis in the current trial and for future use in ancillary studies, if applicable	N/A	Biological specimens will not be collected

It is strongly recommended that this checklist be read in conjunction with the SPIRIT 2013 Explanation & Elaboration for important clarification on the items. Amendments to the protocol should be tracked and dated. The SPIRIT checklist is copyrighted by the SPIRIT Group under the Creative Commons "Attribution-NonCommercial-NoDerivs 3.0 Unported" license.

For peer review only

Supplementary file 2: Baseline questionnaires for healthcare professionals and facilities

Study Questionnaire for Healthcare Professionals

Research Assistant: ____________________ Code: [ ]

Region: ______________________________ Code: [ ]

Health Facility Name: ____________________________ Code: [ ]

Date of Interview: ____/_____/_____

Adverse drug reaction (ADR) is an unwanted or harmful reaction experienced following the administration of a drug or combination of drugs under normal doses/conditions of use & is suspected to be related to the drug. An ADR might require the drug to be discontinued or the dose reduced.

SECTION A: DEMOGRAPHICS

1. Gender:
   [1] Male
   [2] Female
2. Age (*in completed years*): ______
3. Do you own a working smartphone?
   [1] Yes
   [2] No

   3a) If ***YES*** to **Q3**, are you willing to join a WhatsApp group? [1] Yes [2] No

   3b) If ***YES*** to **Q3a**, can you share your WhatsApp number? _____________

4. Do you own a simple working mobile phone *(not a smartphone)*?
   [1] Yes
   [2] No
5. Highest Education Level:
   [1] Certificate
   [2] Diploma
   [3] Bachelors
   [4] Masters
   [5] Other, (*specify*)______________
6. Professional experience (*Years*): [ ]
7. If less than 1 year in **Q5**, state number of *completed months* _____
8. Do you use any of the following? (***Tick all that apply***)
   [1] WhatsApp
   [2] Facebook
   [3] Twitter
   [4] Other, (*specify*) _____________
9. Professional Cadre:
   [1] Physician
   [2] Medical Officer
   [3] Pharmacist
   [4] Nurse/Midwife
   [5] Clinical Officer
   [6] Pharmacy Technician
   [7] Lay Counsellor
   [8] Expert Client
   [9] Other, (*specify*) _____________
10. Health Facility Type:
    [1] Public
    [2] Private Not-for-Profit
    [3] Private for-Profit
11. Health Facility Status:
    [1] Regional Referral Hospital
    [2] Hospital
    [3] Health Centre IV
    [4] Health Centre III
    [5] Health Centre II
    [6] Private Clinic
    [7] Other, (*specify*) _____________

For peer review only

SECTION B: DOLUTEGRAVIR-LINKED AND ISONIAZID-LINKED ADRS

Please, complete the questionnaire by indicating the appropriate responses.

1. What is the approximate number of HIV-positive patients you see per day? ______

Dolutegravir-linked adverse drug reactions

2. Dolutegravir-based antiretroviral therapy was recently rolled out in the treatment of HIV-positive patients, have you ***ever encountered*** any dolutegravir-linked adverse drug reactions (ADRs) amongst the HIV-positive patients in your care?
   [1] Yes [2] No [9] I don't know

3. In the past 4-weeks, have you ***suspected*** any dolutegravir-linked ADRs?
   [1] Yes [2] No (***Skip to 5***) [9] I don't know (***Skip to 5***)

4. If ***YES*** to **Q3**, how many cases of dolutegravir-linked ADRs have you ***suspected*** in the past 4-weeks? _____

5. In the past 4-weeks, have you ***received*** any ***patient-complaints*** of dolutegravir-linked ADRs?
   [1] Yes [2] No (***Skip to 7***) [9] I don't know (***Skip to 7***)

6. If ***YES*** to **Q5**, how many ***patient-complaints*** of dolutegravir-linked ADRs have you ***received*** in the past 4-weeks? ______

7. Briefly describe the most recent ***dolutegravir-linked*** ADR you have encountered giving details on ***patient age***, ***sex***, ***approximate ADR-date, details of the adverse drug reaction***, ***severity*** *(mild, moderate, severe)*, ***seriousness*** *(led to or prolonged hospitalization, caused incapacitation, led to any other medically significant condition or death)*, ***clinical outcome*** & ***action taken***:

   Patient age [ ] years; **sex** [M or F]; **approximate ADR-date** [*mm/yyyy*: __ / ____]

   ADR description (*include severity, seriousness, clinical outcome and action taken*)____
   ____
   ____
   ____
   ____
   ____

Isoniazid preventive therapy-linked adverse drug reactions

8. HIV-positive patients are at a higher risk of dying when they develop active tuberculosis (TB). During the past one year, Ministry of Health scaled up the roll-out of isoniazid preventive therapy (IPT) among HIV-positive patients to significantly reduce these patients' risk of developing active TB. IPT, however, has been associated with ADRs. Have you ***ever encountered*** any IPT-linked ADRs amongst the HIV-positive patients in your care?
   [1] Yes [2] No [9] I don't know

9. In the past 4-weeks, have you ***suspected*** any isoniazid preventive therapy-linked ADRs?
   [1] Yes [2] No (***Skip to 11***) [9] I don't know (***Skip to 11***)

For peer review only - http://bmjopen.bmj.com/site/about/guidelines.xhtml

10. If ***YES*** to **Q9**, how many isoniazid preventive therapy-linked ADRs have you ***suspected*** in the *past 4-weeks*? ______

11. In the past 4-weeks**,** have you ***received*** any ***patient-complaints*** of isoniazid preventive therapy-linked ADRs?

    [1] Yes [2] No (***Skip to 13***) [9] I don't know (***Skip to 13***)

12. If ***YES*** to **Q11**, how many ***patient-complaints*** of isoniazid preventive therapy-linked ADRs have you ***received*** in the past 4-weeks? ______

13. Briefly describe the most recent ***isoniazid-linked*** ADR you have encountered giving details on ***patient age***, ***sex***, ***approximate ADR-date, details of the adverse drug reaction***, ***severity*** *(mild, moderate, severe)*, ***seriousness*** (*led to or prolonged hospitalization, caused incapacitation, led to any other medically significant condition or death*), ***clinical outcome*** & ***action taken***:

Patient age [ ] years; **sex** [M or F]; **approximate ADR-date** [*mm/yyyy*: __ / ____]

ADR description (*include severity, seriousness, clinical outcome and action taken*)____

____

____

____

____

____

14. In the past 6-months, have you ***reported*** any suspected ADRs experienced by the HIV-positive patients under your care?

    [1] Yes [2] No (***Skip to Q23***) [9] I don't know (***Skip to Q23***)

15. If ***YES*** to **Q14**, how many ***ADR reports*** did you make? [ ][ ]

16. If ***YES*** to **Q14**, which drug classes were suspected? (***Tick all that apply***)

    [1] Antiretroviral therapy

    [2] Isoniazid preventive therapy

    [3] Antituberculosis therapy

    [4] Other, (*specify*)______________________________

17. If ***YES*** to **Q14**, which of the following drugs were implicated? (***Tick all that apply and mention number of reported ADRs in past 4-weeks for options [1] & [2]***)

    [1] Dolutegravir: *Number of ADRs* ***reported*** *in the past 4-weeks* ______

    [2] Isoniazid: *Number of ADRs* ***reported*** *in the past 4-weeks* ______

    [3] Other, (*specify*) _______________

For peer review only - http://bmjopen.bmj.com/site/about/guidelines.xhtml

18. If ***YES*** to **Q14**, to whom did you report the most recent ADR? (***Tick all that apply***)
   [1] District Health Officer
   [2] Health Management Information System
   [3] Immediate Supervisor
   [4] National Drug Authority - National Pharmacovigilance Center
   [5] Other, (*specify*)___________________

19. If ***YES*** to **Q14**, how did you report the most recent suspected ADR? (***Tick all that apply***)
   [1] Verbally
   [2] Written paper report
   [3] Written online/website report
   [4] Other, (*specify*) ___________________

20. If ***YES*** to **Q14**, did you get feedback on the ADR(s) you reported?
   [1] Yes [2] No [9] Not sure

21. If ***YES*** to **Q20**, what form of feedback?
   ____
   ____

22. In special situations, the National Drug Authority may need additional information about reported ADRs. Would you be willing to answer further questions after sending a report?
   [1] Yes [2] No [9] I don't know

Herbal Medicine Use

23. In the past 6-months, have you suspected that some of your patients use herbal medicines together with ARVs? [1] Yes [2] No

24. If YES to **Q23**, have you inquired from the patients whether they use herbal medicines together with ARVs? [1] Yes [2] No

25. In the past six months, have any of your patients reported to you that they use herbal medicines together with ARVs? [1] Yes [2] No

26. Have any of your patients presented with ADRs likely to be linked to the use of herbal medicines? [1] Yes [2] No

We appreciate the time you've taken to respond to this survey. Thank you!

For peer review only - http://bmjopen.bmj.com/site/about/guidelines.xhtml

Health Facility Data

Research Assistant: ____________________ Code: [ ]

Region: ______________________________ Code: [ ]

Health Facility Name: ____________________________ Code: [ ]

Date of Enrolment of Health Facility: ____/_____/_____

Average daily number of ART patient visits at the Health Facility: [ ][ ][ ]

Total number of patients on dolutegravir-containing antiretroviral therapy [ ][ ][ ][ ]

Total Number of patients on Isoniazid Preventive Therapy [ ][ ][ ][ ]

Number of healthcare professionals at the Health Facility:

- Medical Doctors [ ][ ]
- Pharmacists [ ][ ]
- Clinical Officers [ ][ ]
- Nurses/Midwives [ ][ ]
- Pharmacy Technicians [ ][ ]
- Village Health Teams [ ][ ]
- Lay Counsellors [ ][ ]
- Expert Clients [ ][ ]
- Other (*specify*) ……………………… [ ][ ]

Does the Health Facility provide health workers with internet access?

1. No   2. Yes   9. Unknown

If YES, describe the form of internet access provided (*wireless, wired, data etc.*):

____

____

Is mobile telephone network connectivity available at the Health Facility?

1. No   2. Yes   9. Unknown

If YES, describe the reliability of mobile telephone network connectivity:

____

____

For peer review only - http://bmjopen.bmj.com/site/about/guidelines.xhtml

Informed Consent Form

Purpose of the study: Experts from National Drug Authority and Makerere University College of Health Sciences are working together to improve the rate and quality of reporting of adverse drug reactions linked to the newly rolled-out dolutegravir-containing antiretroviral therapy and scaled up isoniazid preventive therapy to reduce the risk of tuberculosis in HIV-positive people. This project focuses on the involvement of healthcare professionals in the active drug safety monitoring and management of adverse drug reactions to these drugs and how this system can be improved.

Procedures: In this survey, we ask you to recall and describe the most recent suspected and/or reported adverse drug reactions (ADRs) linked to the use of dolutegravir-containing antiretroviral therapy and isoniazid preventive therapy among the HIV-positive people in your care; and any ADRs you have suspected in the past 4-weeks and/or ADR-complaints you have received from HIV patients in the past 4-weeks. The survey is anonymous and will take no more than 20-30 minutes to complete. After the interview, we shall provide you with the tools you will use to report any adverse drug reactions you encounter, particularly those linked to the use of dolutegravir and isoniazid preventive therapy. You will be contacted by phone at the end of the study to obtain feedback from you about your experience while using the tools for reporting adverse drug reactions. You could even be invited to participate in a group or individual in-depth discussion to share your experiences.

Time compensation: We greatly appreciate your time and opinion. The time you spend filling in this questionnaire will be compensated at a rate of UGX 20,000.

Confidentiality: Any information obtained in this study that can identify you will remain confidential and be disclosed only with your permission or as required by law. Otherwise, confidentiality will be maintained because the self-completed questionnaire is anonymous. We will not request for your name in the information we get from this study.

Participation and withdrawal: Participation is voluntary and you can withdraw at any time.

Identification of Investigators: If you have any questions or concerns about the research, please feel free to contact: Dr. Ronald Kiguba, Principal Investigator, Department of Pharmacology & Therapeutics, School of Biomedical Sciences, Makerere University College of Health Sciences, Tel: +256712840683 & email: kiguba@yahoo.co.uk

Rights of participants: The School of Biomedical Sciences Research & Ethics Committee and the Uganda National Council for Science & Technology (UNCST) have reviewed my request to conduct this project. If you have any concerns about your rights in this study, please contact Dr. Erisa Mwaka Tel: +256 752575050 & email: erisamwaka@gmail.com OR UNCST on +256 414 705500 / +256 312 314800

I understand the procedures described above. My questions have been answered to my satisfaction, and I agree to participate in this study. I have been given a copy of this form.

Signature of participant: ……………………………… Date: ……………………………………

Signature of research assistant: ……………………….. Date: ……………………………………

For peer review only
